# Repurposed AT9283 triggers anti-tumoral effects by targeting MKK3 oncogenic functions in Colorectal Cancer

**DOI:** 10.1186/s13046-024-03150-4

**Published:** 2024-08-20

**Authors:** Valentina Piastra, Federica Ganci, Andrea Sacconi, Angelina Pranteda, Matteo Allegretti, Roberta Bernardini, Martina Serra, Barbara Lupo, Emanuela Dell’Aquila, Gianluigi Ferretti, Edoardo Pescarmona, Armando Bartolazzi, Giovanni Blandino, Livio Trusolino, Gianluca Bossi

**Affiliations:** 1 Translational Oncology Research Unit, Department of Diagnostic Research and Technological Innovation, IRCCS - Regina Elena National Cancer Institute, Via Elio Chianesi, Rome, 53 - 00144 Italy; 2 Department of Science, University Roma Tre, Rome, Italy; 3 Interdepartmental Center for Comparative Medicine, Alternative Techniques and Aquaculture (CIMETA), University of Rome Tor Vergata, Rome, Italy; 4 Candiolo Cancer Institute - FPO IRCCS, Candiolo, Turin, Italy; 5 Department of Oncology, University of Torino, Candiolo, Turin, Italy; 6 Second Division of Medical Oncology, IRCCS - Regina Elena National Cancer Institute, Rome, Italy; 7 First Division of Medical Oncology, IRCCS - Regina Elena National Cancer Institute, Rome, Italy; 8 Department of Pathology, IRCCS - Regina Elena National Cancer Institute, Rome, Italy; 9 Pathology Research Laboratory, St Andrea University Hospital, Rome, Italy

**Keywords:** MKK3/p38MAPK, Drug repurposing, Aurora kinase A, Target therapy, Colorectal cancer (CRC)

## Abstract

**Background:**

Colorectal cancer (CRC) is the third most common type of cancer and the second leading cause of cancer-related deaths worldwide, with a survival rate near to 10% when diagnosed at an advanced stage. Hence, the identification of new molecular targets to design more selective and efficient therapies is urgently required. The Mitogen activated protein kinase kinase 3 (MKK3) is a dual-specificity threonine/tyrosine protein kinase that, activated in response to cellular stress and inflammatory stimuli, regulates a plethora of biological processes. Previous studies revealed novel MKK3 roles in supporting tumor malignancy, as its depletion induces autophagy and cell death in cancer lines of different tumor types, including CRC. Therefore, MKK3 may represent an interesting new therapeutic target in advanced CRC, however selective MKK3 inhibitors are currently not available.

**Methods:**

The study involved transcriptomic based drug repurposing approach and confirmatory assays with CRC lines, primary colonocytes and a subset of CRC patient-derived organoids (PDO). Investigations in vitro and in vivo were addressed*.*

**Results:**

The repurposing approach identified the multitargeted kinase inhibitor AT9283 as a putative compound with MKK3 depletion-mimicking activities. Indeed, AT9283 drops phospho- and total-MKK3 protein levels in tested CRC models. Likely the MKK3 silencing, AT9283 treatment: *i*) inhibited cell proliferation promoting autophagy and cell death in tested CRC lines and PDOs; *ii*) resulted well-tolerated by CCD-18Co colonocytes; *iii)* reduced cancer cell motility inhibiting CRC cell migration and invasion; *iv)* inhibited COLO205 xenograft tumor growth. Mechanistically, AT9283 abrogated MKK3 protein levels mainly through the inhibition of aurora kinase A (AURKA), impacting on MKK3/AURKA protein–protein interaction and protein stability therefore uncovering the relevance of MKK3/AURKA crosstalk in sustaining CRC malignancy in vitro and in vivo.

**Conclusion:**

Overall, we demonstrated that the anti-tumoral effects triggered by AT9283 treatment recapitulated the MKK3 depletion effects in all tested CRC models in vitro and in vivo, suggesting that AT9283 is a repurposed drug. According to its good tolerance when tested with primary colonocytes (CCD-18CO), AT9283 is a promising drug for the development of novel therapeutic strategies to target MKK3 oncogenic functions in late-stage and metastatic CRC patients.

**Supplementary Information:**

The online version contains supplementary material available at 10.1186/s13046-024-03150-4.

## Background

CRC is the third most common malignancy worldwide [1] and the second most common cause of cancer-related death in the United States, with a high incidence rate in individuals younger than 50 years [2]. Although early-stage CRCs are mainly treated with surgery, chemotherapy still constitutes the first-line treatment for unresectable metastatic disease. Current therapeutic treatments are based on fluoropyrimidine 5-fluorouracil (5-FU) as a single agent or in multi-chemotherapy regimens, such as FOLFOX (leucovorin, 5-FU, and oxaliplatin), FOLFIRI (leucovorin, 5-FU, and irinotecan), and CAPOX/XELOX (capecitabine and oxaliplatin) [3]. Nevertheless, advanced stage and metastatic CRC is still a fatal disease with a survival rate near to 10%, making the identification of new molecular mechanisms to target an imperative need for the development of more selective and efficient therapeutic strategies [4].

MKK3 is a dual-specificity threonine/tyrosine protein kinase that is activated through phosphorylation at specific serine and threonine residues (Ser189/Thr222) by MKKK proteins (MEKK 1–4) [5] in response to cellular stress and inflammatory stimuli, regulating a plethora of biological processes. Previous studies revealed the MKK3 relevance in supporting tumor malignancy, indeed its depletion induces autophagy and cell-death in cancer lines of different tumor types, including CRC [6–8]. High MKK3 staining correlated significantly with advanced CRC tumor stages when investigated in a retrospective CRC tissue microarrays (TMAs) cohort [8]. Interestingly, analyses of protein‒protein interaction network of cancer-associated genes (Onco-PPI) [9] revealed MKK3, beyond its canonical function as p38MAPK upstream activator, as one of the major hub proteins complexing with several key players critically involved in the regulation of cellular proliferation and metabolism including MYC [9]. Indeed, MKK3 stabilizes MYC protein, enhances its transcriptional activity and increases expression of MYC-regulated genes, therefore suggesting the MKK3/MYC complex disruption as a new targeted therapy to thwart MYC oncogenic programs [10]. We recently demonstrated the MKK3/MYC crosstalk as a novel molecular mechanism thwarting the therapeutic efficacy of BRAF inhibitors (dabrafenib) in BRAF-mutated CRC, ultimately leading to dabrafenib resistance [11]. Furthermore, MKK3 sustains the epithelial-mesenchymal transition (EMT) and tumor angiogenesis by driving the expression of a specific set of genes [11–15], and supports the malignancy of specific tumor types (as reviewed in [12]). By contrast, MKK3 appears dispensable in normal tissues, as its knockdown resulted well-tolerated in primary cultures [6, 8], and mice MKK3 knockout are viable and fertile [16]. The overall reported features make MKK3 an attractive and promising target for the development of new therapeutic approaches.

MKK3 inhibitors, specifically targeting the protein kinase, are not currently available, and the identification and validation of new drugs targeting the MKK3 functions would require time-consuming and expensive medicinal chemistry before their credentialing as valuable alternatives in clinical practice. In this study, through a drug-repurposing strategy [17], we deemed to identify FDA-approved compounds with MKK3-knockdown mimicking activities (MKDMA) and known clinical/preclinical testing and thus quickly translatable in the clinical practice [18]. Results identified AT9283 (1-cyclopropyl-3-(3-(5-(morpholinomethyl)-1H-benzo[d]imidazol-2-yl)-1H-pyrazol-4-yl) urea) as a putative MKDMA. AT9283 is a multi-targeted inhibitor of Aurora A, Aurora B, JAKs, Abl, and Flt3 kinases, with potent inhibitory activity against aurora kinase when tested in phase I/II clinical trials in adults and children with solid and hematologic malignancies [19–26]. Herein, we investigated and validated AT9283 as a putative MKDMA by using different in vitro and in vivo experimental models and shed new lights on the relevance of the MKK3/AURKA crosstalk in sustaining their own protein stability and activity and thus CRC malignancy. Overall, our data suggest that AT9283 is a repurposed drug for targeting the MKK3 oncogenic functions in CRC.

## Methods

### Cell lines

Authenticated ECACC CRC lines HT29 (ECACC 91072201), COLO205 (ECACC 87061208), COLO320DM (ECACC 87061205), SW480 (ECACC 87092801), SW620 (ECACC 87051203), and HCT116 (ECACC 91091005); authenticated ATCC primary human colonocyte CCD-18Co (CRL-1459); as well as the engineered COLO205 and HT29 sh/MKK3 and sh/scr sublines [8] were regularly monitored for mycoplasma contamination every 2 months by PCR [27], and only mycoplasma-free cells were used for studies. All lines were used for no more than 15 passages. COLO205 and COLO320DM cells were grown in RPMI (31,870, Gibco, Paisley, Scotland, UK); HT29 and HCT-116 cells were grown in McCoy’s (12-688F, Lonza, Burton on Trent, UK); SW480 and SW620 cells were grown in L15 (BE12-700F, Lonza); and CCD-18CO were grown in DMEM (M2279 Sigma-Aldrich, Saint Louis, MO, USA). All culture media were supplemented with 10% heat-inactivated FBS (10,099–141, Gibco, Paisley, Scotland, UK), 2 mM L-glutamine (17-606E, Lonza) and 100 U/mL penicillin/streptomycin (DE17-602E Gibco). All cells were incubated at 37 °C in humidified conditions with 5% CO2.

### CRC-PDOs

CRC1502 (CRC1502LMO0A), CRC1757 (CRC1757LMO0A), and CRC0322 (CRC0322LMO0A) (Table 1), were kindly provided by Dr. Trusolino (Candiolo Cancer Institute Torino, Italy) [28]. PDOs were cultured in extracellular matrix hydrogel (Cultrex growth factor reduced PathClear Basement Membrane Extract, type 2: R&D Systems 3533–010-02) and DMEM:F12 (D6421, Sigma-Aldrich) supplemented with 1.0% penicillin/streptomycin, 1.0% B27 Supplement (17,504–044, Gibco), 1.0% N2 Supplement (17,502–048, Gibco), 1.0 mM N-acetylcysteine (A9165, Sigma-Aldrich), 2 mM L-glutamine, 100 U/mL penicillin/streptomycin, 20 ng/ml rEGF (E9644, Sigma-Aldrich).

**Table 1:** Descriptive features of selected Patient-derived organoids

CASE	CRC1757LMO0A	CRC1502LMO0A	CRC0322LMO0A
**KRAS**	*wt*	*G12D*	*wt*
**NRAS**	*wt*	*wt*	*wt*
**BRAF**	*V600E*	*wt*	*wt*
**Age at collection**	*52*	*68*	*56*
**sex**	*F*	*M*	*NA*^*a*^
**Stage at first diagnosis**	*IV*	*IV*	*NA* ^*a*^
**Site of primary**	*Rectum*	*Rectum*	*Rectum*
**T**	*3*	*3*	*3*
**N**	*2B*	*1A*	*2*
**M**	*1*	*1*	*1*
**N° of metastases**	*2*	*4*	*NA* ^*a*^
**Site M**	*LIVER, LUNG*	*LIVER, LIMPHONODES*	*NA* ^*a*^
**Treatments**	*FOLFIRINOX*	*NA*^*a*^	*XELODA* + *FOLFOX* + *BEVACIZUMAB*

^a^Not Assessed

### Gene expression profiling

HT29-sh/scr and sh/MKK3 sublines were plated (3.0 × 10^5^ cells/60 mm dish), and treated 24 h later with 1 μg/ml doxycycline (DOX) (D9891 Sigma‒Aldrich). Sixty h later cells were collected and total RNA extracted from biological replicates with miRNAeasy® kit (Qiagen, Chatsworth, CA) following the manufacturer’s instructions. RNA concentration and purity were assessed with Nanodrop TM1000 spectrophotometer (Wilmington, DE, USA). Reverse transcription and RT‒qPCR were performed with an MMLV RT assay and Sybr Green® or TaqMan Assays (Applied Biosystems, Foster City, CA, USA) according to the manufacturer's protocol. H3, GAPDH, GUSB, RPL19, RNU2 and ACTIN were used as endogenous controls to normalize gene expression. The Affymetrix gene expression data (array HTA_2.0) were background adjusted and quantile normalized, and gene expression values quantified with robust multiple-array average (RMA) procedure, and duplicates were averaged. Probes not detected in all the samples were removed. The summarized signals were log2-transformed and quantile normalized. Significantly modulated genes between different conditions were identified with a distribution fold change test (DFC) and a permutation test, adjusting for multiple comparisons. DataSet are available at Gene Expression Omnibus (GEO) with ID GSE262634. 

### Analysis of TCGA COADREAD data

Gene expression data were normalized to tumor samples from The Cancer Genome Atlas (TCGA) Data Analysis Center (http://gdac.broadinstitute.org/) using the Firehose stddata__2016_01_28 dataset provided by the Broad Institute of MIT and Harvard (10.7908/C11G0KM9). Disease-free survival (DFS) and overall survival (OS) analyses were performed with Kaplan‒Meier curves. Differences between survival curves were assessed using the log-rank test. The impact of clinical variables on survival was evaluated with a multivariate Cox proportional hazards regression model. Patients were categorized into high- and low-signal intensity groups based on positive and negative z scores unless otherwise specified. The prognostic relevance of the identified signatures was assessed by the average expression of selected gene sets.

### Perturbation-driven gene expression dataset

Specific gene signatures for drug prediction were employed utilizing the CMap library of Integrated Network-based Cellular Signatures (LINCS) platform version 1 (https://clue.io/) [29]. Scores with absolute values exceeding 90 were deemed to indicate statistical significance, and drugs with scores > 95 were selected. All analyses were conducted using MATLAB R2022a.

### Drug treatments

CRC lines and colonocytes were plated (5 × 10^3^ cells/96 well) and treated 24 h later either with (a) AT9283 (896,466–04-9, Cayman Chemical, E. Ellsworth Road Ann Arbor, MI, USA), (b) alisertib (1,028,486–01-2, MedChemExpress) or (c) barasertib (AZD1152-HQPA, Selleckchem Bioactive Compounds Expert, Canada). Cells were analysed 96 h later by MTT Assay Kit (ab211091, Abcam) following the manufacturer’s guidelines, and absorbance quantified on a MULTISKAN EX 200–240 V (Thermo Scientific Carlsbad, CA, USA) at 540 nm. For trypan blue exclusion dye assay, COLO205 and HT29 cells were plated (5 × 10^4^ cells/24-well plates) and treated 24 h later with the respective AT9283 IC_50_ (15 nM, 100 nM), and cells analysed at specific time points post-treatment (24–72 h). Results were analysed using GraphPad Prism software (8.0.2.263 MSI installer Version 2.0).

For tests with organoids, PDOs seeded on BME hydrogel plug with complete culture medium were treated with increasing doses of AT9283 and daily analysed under microscope (Olympus IX71; 4X magnification) and n.5 images were collected from each replicate and for each tested condition with a Tucsen camera (THC 5.0 ICE). The PDO areas were quantified with ImageJ software, and the results were analysed with Graph Pad Prism software.

### Colorimetric autophagy assay

Twenty-four hours after plating, COLO205 (2 × 10^6^ cells/60 mm dish) or HT29 (1.5 × 10^5^ cells/35 mm dish) cells were left untreated or treated either with AT9283 72 h (15 nM and 100 nM) or with rapamycin (0.5 µM) and chloroquine (10 µM) 24 h. After treatments, cells were stained with Autophagy Detection Kit (Enzo Life Sciences CYTO-ID^R^) following manufacturer’s guidelines, and stained cells analysed with Airy-Scan confocal laser scanning microscope (LSM880 ZEISS, Carl Zeiss AG, Oberkochen, Germany) at 60X magnification.

### Inducible MKK3 depletion

To deplete endogenous MKK3, engineered COLO205 and HT29-sh/MKK3 and -sh/scr sublines were seeded (3 × 10^5^/60 mm dish) and shRNA expression induced with 1 μg/mL DOX, delivered into the culture media and renewed every 3 days.

For PDO engineering, organoids were seeded as single cells (1000 cells/24-well plate) and transduced with a TET-inducible lentivirus-based system carrying either an shRNA sequence specific to MKK3 (sh/MKK3) or a control sequence (sh/scr) at an MOI of 20, as previously described [6–8]. The day after, derived sh/MKK3 and sh/scr PDOs were collected and cultured with the BME hydrogel as described above. To induce sh/RNA expression, engineered PDOs sh/MKK3 and sh/scr were cultured with DOX (1 μg/ml) and renewed every 3 days. To assess the effects on cell viability, 72-h DOX-pre-treated PDOs-sh/MKK3 and -sh/scr were seeded as single cells (15,000 cells/96 wells) and further treated for 72 h with DOX (1 μg/ml) before MTT assays. The results were analysed using GraphPad Prism software.

### AURKA knockdown

COLO205 and HT29 cells were seeded (1 × 10^5^/60 mm dish) and co-transfected with control (si/CT) or AURKA-specific stealth RNAi (80 nM) oligos (AURKAHSS186149, 10,620,318–431521 CO2, Invitrogen Carlsbad, CA, USA) and (AURKAHSS186149: 10,620,319–431777 B06, Invitrogen Carlsbad, CA, USA) with Lipofectamine RNAi MAX Reagent (MAN0007825, invitrogen) according to manufacturer’s specifications. Forty-eight hours later cells were collected for WB analyses whereas 72 h later for MTT assay. AURKA knockdown efficiency routinely ranged from 80 to 90% as protein level reduction when compared to control cells (Suppl. Fig. 6A).

#### Ectopic MKK3 expression

COLO205 cells were seeded (2 × 10^5^ cells/6-well plate) and transfected either with empty (pcDNA3) or MKK3-encoding (pcDNA3-HA-MKK3) [30] vectors with Lipofectamine LTX and Plus reagent (15,338, invitrogen) according to the manufacturer’s specifications. All experiments were performed within 3 passages after transfection.

#### Western blot analyses

Protein lysates in RIPA buffer [(150 mM NaCl, 1% Triton X, 0.25% sodium deoxycholate, 0.1% SDS, 50 mM Tris/HCl, 2 mM EDTA) + complete mini protease inhibitor cocktail (#11,836,153,001, Roche, Basel, Switzerland) + PMSF (1 mM) + Aprotinin (19 μg/mL) + NaF (50 mM) + DTT (50 mM) + Na₃VO₄ (1 mM)] were resolved on an SDS/PAGE precast gel (XP04205BOX, 4–20% Tris–Glycine Gel Novex™ WedgeWell™, Invitrogen) and transferred to PVDF membranes (iBlot 2 PVDF: IB24001, IB24002, Invitrogen). The membranes were blocked in 5% BSA (A1391, AppliChem, Darmstadt, Germany) or nonfat-dried milk (M7409, Sigma Aldrich) and incubated overnight at + 4 °C with the following primary antibodies:

**Table:** 

Protein	Dilution	Antibodies
pMKK3/6 ser189/207	1:1000	9231, Cell Signaling Technology
MKK3	1:1000	H00005606-M02, Abnova
p-AKT Ser473	1:2000	D9E, Cell Signaling Technology
AKT	1:1000	C67E7, Cell Signaling Technology
p-mTOR Ser2481	1:1000	2974, Cell Signaling Technology
mTOR	1:1000	7C10, Cell Signaling Technology
p62/SQSTM1	1:1000	sc-28359, Santa Cruz Biotechnology, inc
LC3BI/II	1:1000	L7543, Sigma‒Aldrich
PARP	1:500	9542, Santa Cruz Biotechnology, inc
Cleaved PARP Asp214	1:1000	D64E10, Cell Signaling Technology
Aurora kinase A	1:1000	D3E4Q, Cell Signaling Technology
E-cadherin	1:5000	20,874–1-AP, Proteintech
SLUG	1:1000	A1057, ABclonal
Vimentin	1:1000	PR3776, ABCAM
Phospho-Histone H3 (Ser10)	1:1000	9701, Santa Cruz Biotechnology
GAPDH	1:10,000	G8796, Sigma Aldrich
β-Actin	1:1000	4970, Cell Signaling Technology

Appropriate anti-mouse (1,706,516, Bio-Rad, Hercules, CA, USA) and anti-rabbit (1,721,019, Bio-Rad) HRP-conjugated secondary antibodies were used, immunocomplexes were visualized with enhanced chemiluminescence (ECL) (RPN2106, GE Healthcare, Chicago, IL, USA), and bands were quantified by densitometry with ImageJ software.

#### RT‒PCR analyses

Engineered COLO205 and HT29-sh/MKK3 and sh/scr sublines were plated (3 × 10^5^ cells/60 mm dish) and incubated with 1 μg/ml DOX, and 120 h, 144 h later cells collected. COLO205 and HT29 cells were plated (3 × 10^5^ cells/60 mm dish) and left untreated or treated with AT9283 (15 nM and 100 nM) for 24 h before harvesting. Total RNA was extracted with TRIzol Reagent (AMBION Life Technologies, Van Allen Way, Carlsbad, CA, USA) and reverse-transcribed (2 μg) with a FastGene Scriptase Basic cDNA Kit (NIPPON Genetics Europe, Duren, Germany) following the manufacturer’s guidelines. The generated cDNAs were analysed by q-PCR (PB20, PCR Biosystems 2 × qPCR SYBR Green Mix Separate ROX) with the following primer sets:

**Table:** 

Gene	Forward 5’ > 3’	Reverse 5’ > 3’
MKK3	*CTACATGGCCCCTGAGA*	*TCCAGACGTCGGACTTGACAGGAT*
UPK3BL	*AGCAGCCACAACATCTCTGA*	*GTTGGTTGGCAGTGGGTATC*
PYCR2	*AAGCTCCTCACAAGAAGCCT*	*TCGCATTGCTTGGTCTGAAC*
PPP1R3E	*CAAACCCCACCATCTGCAAA*	*AGTGTTCCCCGTGTCAGATT*
SNCG	*CCATCCCCTCCTAGCACAAG*	*AGCAGCATAAGTGGGGTCAG*
ARHGAP1	*CGACCAGTACAATGAGCTGC*	*AGGAAACGAAGCACCTGGTA*
CAPRIN2	*CCAGTGCCAACTGCCATCTA*	*TGGCTGAGTAGGTCTGGGAA*
ATP6V1B1	*CTACAGGACTGTGTGCAGCG*	*GAACAATCGCCTTGGTGCC*
F2RL1	*GCGATCTTCTGCCATGGATG*	*GTCGATGCAGCTGTTAAGGG*
GSPT1	*TGGTACTTGTCTTGGGCCTT*	*TGAGATTACCAGCACAGCCA*
NAT1	*AAGAAATCGGGGTGGATGGT*	*CCAAACCCAGCATCGACAAT*
ZEB1	*GCAGTCCAAGAACCACCCTT*	*GGGCGGTGTAGAATCAGAGT*
SNAIL	*ACTATGCCGCGCTCTTTCC*	*GTCGTAGGGCTGCTGGAAG*
TWIST1	*TCCGCGTCCCACTAGCAG*	*CTCTGGAAACAATGACATCTAGGTTCT*
ACTIN	*GCTGCCCTGAGGCAATCTT*	*ATGATGGAGTTGAAGGTAGGTTCGT*

Results were normalized to GAPDH housekeeping gene and quantified with respect to relative controls (untreated, sh/scr) set to 1.0 and outcomes reported as relative gene expression, otherwise log2-transformed and reported as log2-fold change.

#### Immunoprecipitation

Twenty-four hours after plating, COLO205 and HT29 cells (2 × 106 cells/150 mm plate) were left untreated or treated with AT9283 (15 nM, 100 nM), and 48 h later harvested and lysed with IP-RIPA buffer (1% Triton, 150 mM NaCl, 50 mM Tris/HCl, 1 mM EDTA) + 50 mM NaF + 1 mM PMSF + 5 mM Na_3_VO_4_ + complete mini protease inhibitor cocktail. Protein lysates (700 μg/sample), precleared for 1 h at 4 °C with Protein G PLUS-A agarose beads (20 μl/sample) (sc-2002 Santa Cruz Biotechnology, Inc.), were incubated on the orbital platform O/N at 4 °C with specific primary antibodies: pMKK3 (1:50, D8E9, Cell Signaling Technology) or Aurora A (1:50, D3E4Q Cell Signaling Technology). The day after protein complexes were incubated for 1 h at + 4 °C with Protein G PLUS-A agarose beads (20 μl/sample) and, after repeated washes with IP-RIPA buffer, samples were denatured for 5 min at 95 °C with IP loading dye (1% SDS, 25 mM Tris/HCl, 10% glycerol, 0.010% bromophenol blue) and resolved in 10% SDS/PAGE. Protein interactions were revealed by western blotting with specific primary antibodies against MKK3 (sc376627 Santa Cruz Biotechnology) and Aurora A (D3E4Q Cell Signaling Technology). Band intensities were quantified by densitometry with ImageJ software.

#### Immunofluorescence

Twenty-four hours after plating, COLO205 and HT29 cells (1.0 × 10^5^ cells/6-well plate) were left untreated or treated for 48 h with AT9283 (15 nM, 100 nM), whereas derived sh/scr and sh/MKK3 sublines were pretreated for 96 h with DOX (1 μg/ml). Thereafter, cells were fixed for 10 min at RT with 1X PBS + 1% formalin (Sigma‒Aldrich), permeabilized with 1X PBS + 0.1% TRITON (TRITON 100X, Sigma‒Aldrich), blocked 20 min at RT with 1X PBS/NaN_3_ + 10% FBS (#10,099–141, Gibco), incubated 1 h at RT either with Aurora A (1:100, D3E4Q, Cell Signaling Technology), MKK3 (1:100, sc376627, Santa Cruz Biotechnology) or α-Tubulin (1:25, 2144, Cell Signaling Technology) primary antibodies diluted in 1X PBS/NaN_3_ + 1.5% FBS, and 1 h at RT either with Alexa Fluor 488-conjugated AffiniPure goat anti-rabbit IgG (1:400, 111–545-003, Jackson ImmunoResearch Carlsbad, CA, USA) or Alexa Fluor 594-conjugated AffiniPure goat anti-mouse IgG (1:400, 115–585-003, Jackson ImmunoResearch) secondary antibodies diluted in 1X PBS/NaN_3_ + 5% FBS. After and nuclear staining, 15 min at RT with 4′,6-diamidino-2-phenylindole (DAPI) (1 mg/ml, Thermo Fisher Scientific), slides were mounted and analysed with an Airy-Scan confocal laser scanning microscope (LSM880 ZEISS, Carl Zeiss AG, Oberkochen, Germany) equipped with 40X or 63X/1.23 NA oil immersion objectives. Images were acquired from 5 different fields from each sample in triplicate and analysed with ImageJ software to quantify the nuclear and cytoplasmic fluorescence intensity of each fluorophore. The intensities of each fluorophore in the nucleus and whole cells were quantified by ImageJ. The relative intensity of cytoplasmic fluorescence (I_C_) was calculated with the formula [I_C_ = (I_Cell_-I_N_) × 100] [31], where I_N_ is the nuclear intensity and I_Cell_ is the cell intensity*.* The data were analysed using GraphPad Prism software.

#### Cell cycle analyses

Twenty-four hours after plating HT29 cells (3 × 10^5^ cells/60 mm dish) were left untreated or treated with AT9283 (100 nM). Forty-eight and 72 h later, the cells were fixed with ice-cold 70% ethanol and stained with propidium iodide-based solution (FxCycle™ PI/RNase Staining Solution cat. F10797, Invitrogen). Cell cycle analyses were performed by ModFit LT software (Verity Software House), disabling auto debris and auto aggregates options, enabling linearity and setting the number of expected cell cycles to 2 (diploid model).

#### Cell migration assay

Cells (8 × 10^4^ cells/insert) were plated on cell culture inserts (3422, COSTAR, Corning Incorporated, 2 Alfred Rd. Kennebunk, ME, USA), and 24 h later migrated cells were fixed 5 min at RT with 1 × PBS + 2% formaldehyde and permeabilized with absolute methanol (V1H03601, Carlo Erba reagents) before staining with Cristal Violet (0.025). Stained inserts were analysed under an optical microscope (Olympus IX71) at 20X magnification. Images were acquired with a Tucsen camera (THC 5.0 ICE), 10 fields/sample were analysed, the number of migrated cells was quantified with ImageJ software, and the results were analysed with Graph Pad Prism software.

#### Wound healing assay

Cells were plated (3 × 10^5^ cells/60 mm dish) and 24 h later challenged with AT9283 or left untreated. Wounds were generated 72 h later at 80% confluence. Cell mobility was assessed 24/48 h later under an optical microscope (Olympus IX71) at 20X magnification, and images were acquired with a Tucsen camera (THC 5.0 ICE) from n.10 fields/sample. Wound gaps were quantified by ImageJ software, and the results were analysed with Graph Pad Prism software. The percentage of wound confluence was calculated with the formula [ (A_0_ – A_T_)/A_0_ × 100], where A_0_ is the gap area recorded at hour 0 and A_T_ is the gap area at time (T).

#### Pre-clinical models and treatments

All in vivo experiments were performed at the Animal Technology Station (University of Tor Vergata) and IFO-IRE animal facility following the EU Directive 2010/63/EU in compliance with institutional guidelines, and regulations and authorization of the appropriate institutional review board (141/2017-PR dated 02/13/2017 and 339/2023-PR dated 04/14/2023). Exponentially growing COLO205 cells were subcutaneously injected (1.0 × 10^6^ cells/mouse) into 45-day-old female nude mice (AthymicNude-Foxn1nu, Envigo, Casatenovo, Italy). After tumor nodule formation (200 mm^3^), mice were randomly assigned to groups (6 mice/group) and treated by gavage (200 µl/mouse) with AT9283 (15 mg/kg) [32–35] or vehicle solution [2% DMSO + 30% PEG300 in water] 5 days a week. Tumor growth was assessed by calliper measurements twice a week, and tumor volumes (TVs) were estimated by the formula TV = a × (b2)/2 [36–38], where a and b are tumor length and width, respectively. At the end of the experimental procedure, all animals were sacrificed, and protein lysates were generated from the tumors, which were excised and analysed by western blotting.

#### Statistical analyses

Where appropriated significance was assessed by Student’s t test, Fisher’s exact test, or one-way or two-way ANOVA. Statistical analysis was evaluated by using GraphPad/PRISM 7 software and the data were displayed as the means ± SDs. Experiments in vitro were performed three times independently. *P* < 0.05 was considered as statistically significant. *P < 0.05; **P < 0.01, ***P < 0.001, and ****P < 0.0001.

## Results

### Drug switching to identify drugs mimicking MKK3 depletion effects

**Fig. 1:**
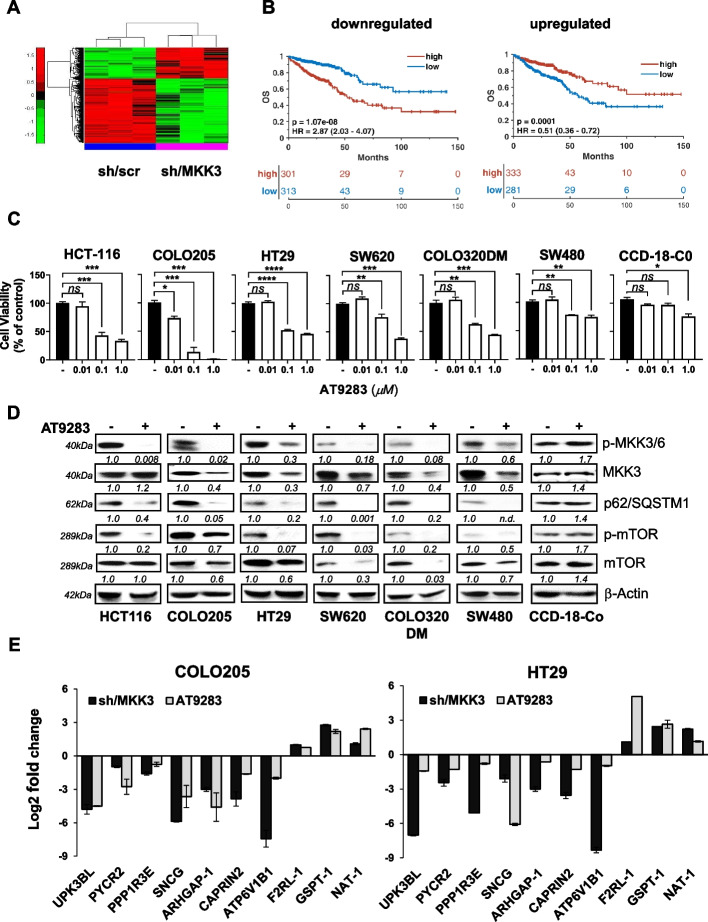
Drug repurposing to identify putative MKDMA drugs. **A** Hierarchical clustering analyses of DEGs accurately distinguished between sh-/scr and sh/MKK3 conditions; **B** Overall survival (OS) data from the TCGA COADREAD cohort of downregulated (n. 52) and upregulated (n. 16) genes in the sh/MKK3 subline compared with the control (sh/scr) subline. Patients were categorized into high- and low-signal intensity groups based on positive and negative z scores, respectively. The log-rank test was used to evaluate differences between survival curves. A multivariate Cox hazard regression model was utilized with adjustments for T, N, M, stage, and MSI status. **C** AT9283 effects on cell proliferation with reported CRC lines and primary colonocytes (CCD-18CO) by MTT assay 72 h later. Outputs were quantified with respect to untreated cells (-) set to 1.0 and eported as mean ± SD. Representative results of three independent experiments in technical triplicates are reported. Significance was assessed with ordinary one-way ANOVA followed by Dunnett’s post-hoc multiple comparisons test: ns, not significant; *p < 0.05; **p < 0.01; ***p < 0.001. **D** AT9283 IC_50s_ treatments: HCT116 and HT29 (50 nM), COLO205 (15 nM), SW620 (100 nM), COLO320DM (885 nM), SW480 (1.6 μM), and CCD-18CO (4 μM) and protein lysates generated 72 h later were analysed by WB with the indicated antibodies. More relevant bands from the same filter at the same exposure length are reported. **E** RNAs was isolated from: I) sh/MKK3 and sh/scr sublines treated 144 h with DOX (1 μg/ml); II) AT9283 treated COLO205 (15 nM) and HT29 (100 nM) cells 48 h. Expression of subset of DEGs was assessed by qPCR. Results were normalized to GAPDH housekeeping gene and quantified with respect to relative controls (untreated; sh/scr) set to 1.0, outcomes were log2-transformed and reported mean ± SD for each DEG. Representative results of three independent experiments in technical triplicates are reported. Significance was assessed with an unpaired Student’s t test: *p* < 0.0001

To identify FDA-approved drugs that target the oncogenic functions of MKK3 in CRC and thus easily translatable into the clinical practice, we implemented a drug repurposing strategy based on transcriptome data. Gene expression perturbations upon MKK3 silencing were investigated with the well-characterized HT29-derived sh/scr and sh/MKK3 sublines [6–8, 11] bearing a well-established inducible RNAi system [6–8, 39]. The engineered sublines were treated in a time-dependent manner with doxycycline (DOX) to determine the time frame at which RNA interference exerted the maximum MKK3 depletion efficiency before the onset of known cytopathic effects (autophagy and cell death), which usually start after 72 h of DOX treatment [6–8]. Efficient MKK3 silencing was achieved after 60 h of DOX treatment (suppl. Fig. 1A), the time at which biological replicates were generated (suppl. Fig. 1B) and gene expression profile analysis performed (see Materials and Methods). Compared to the control (sh/scr), a total of 1799 differentially expressed genes (DEGs) (590 upregulated and 1209 downregulated genes) were revealed in the MKK3-depleted samples (suppl. Fig. 2A). Both PCA and hierarchical clustering analyses demonstrated distinct separation of samples by treatment, indicating that the expression profiles of the identified DEGs accurately distinguished between the 2 sample types (suppl. Fig. 2b and Fig. 1A). The down- and upregulated gene signatures were further analysed for their prognostic relevance in the TCGA-COADREAD patient cohort. These analyses revealed 2 signatures, consisting of 52 unique genes with positive prognostic value (Suppl. Table 1) and 16 genes with negative prognostic value (Suppl. Table 2), for overall survival (OS) (Fig. 1B) or disease-free survival (DFS) (suppl. Fig. 3A, B). The identified upregulated and downregulated prognostic gene signatures were queried separately in the NIH-LINCS database. Thereafter, we selected compounds scoring > 95% that positively modulated the expression of the upregulated genes and negatively modulated the expression of the downregulated genes. AT9283 was among the top-scoring compounds (suppl. Table 3).

### AT9283 recapitulates the effects induced by MKK3 silencing in tested CRC lines

**Fig. 2:**
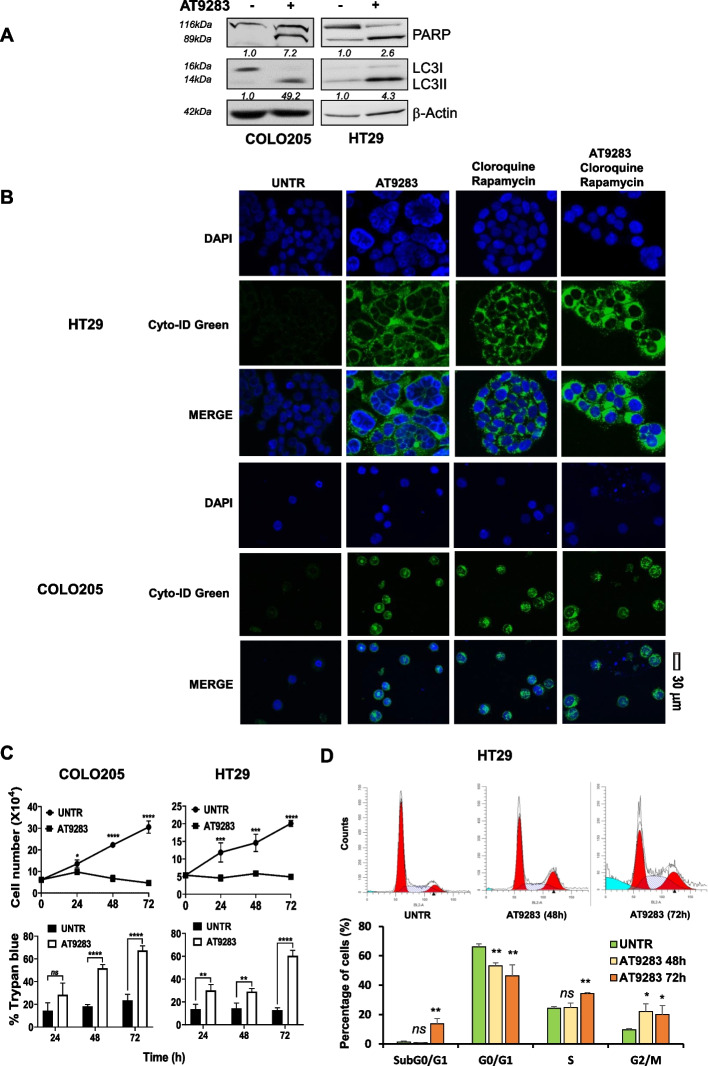
AT9283 induces autophagy and cell death in the tested CRC cells.** A** COLO205 and HT29 cells treated for 72 h with AT9283 at a relative IC_50_ of 15 nM or 100 nM, respectively, were collected, and protein lysates were analysed by western blotting with the indicated antibodies. More relevant bands from the same filter at the same exposure length are reported. **B** CRC cells were treated with AT9283 at the relative IC_50,_ and 72 h later, the cells were stained for autophagolysosome structures and analysed via confocal microscopy (40X magnification). Representative images from three independent experiments with similar results are shown. **C** CRC cells were treated with AT9283 at the relative IC50, and viable and trypan blue-positive cells were quantified at different time points. The representative results of three independent experiments performed in technical triplicates are reported as the means ± SDs. Significance was assessed with ordinary one-way ANOVA followed by Dunnett’s post hoc multiple comparisons test: ns, not significant; *p < 0.05; **p < 0.01; ***p < 0.001; ****p < 0.0001. **D** HT29 cells were left untreated (UNTR) or treated with AT9283 at the IC_50_ (100 nM) for 72 h and analysed by flow cytometry. Representative data from three independent experiments with similar results are reported

To validate AT9283 as a putative MKDMA, primary tests were performed with a panel of CRC lines bearing different mutational backgrounds (COLO205^*BRAFV600E/KRASWT*^, HCT-116^*BRAFWT/KRASG13D*^, HT-29^*BRAFV600E/KRASWT*^, SW620 ^*BRAFWT/KRASG12V*^, SW480 ^*BRAFWT/KRASG12V*^, COLO320DM ^*BRAFWT/KRASWT*^). Treatment with AT9283 significantly inhibited survival in a dose-dependent manner in all CRC lines tested, although to different extents (Fig. 1C). Interestingly, at the molecular level, AT9283 treatment at the half-maximal relative inhibitory concentration (IC_50_) (Suppl. Fig. 4A) decreased the MKK3-phospho and total protein levels to varying degrees (Fig. 1D) and reduced the MKK3 RNA levels (Supplement Fig. 4B) in all the CRC lines tested. Furthermore, AT9283 at the IC_50_ induced autophagy and inhibited PI3K-AKT-mTOR pro-survival signalling in all the CRC lines tested (Fig. 1D). By contrast, AT9283 resulted less effective in primary colonocytes (CCD-18CO) than in CRC lines (Fig. 1C), with no significant effects on MKK3 protein (phospho and total) levels, p62/SQSTM1 autophagic markers or the PI3K/AKT signalling pathway (Fig. 1D). We then explored whether AT9283 might perturb the identified MKK3-dependent gene signatures, and the expression of a randomly selected subset of genes (Suppl. Tables 1 and 2) was investigated by RT‒PCR analyses in the most responsive CRC lines (COLO205, HT29). The AT9283 treatments, like the MKK3 depletion, downregulated and upregulated the expression of selected set of genes in both CRC lines (Fig. 1E). Deeper investigations demonstrated that AT9283 treatments induced: *i*) autophagy, increasing the LC3II/I ratio (Fig. 2A) and the autophagolysosome accumulation (Fig. 2B); *ii)* apoptotic cell death, as assessed by increased PARP cleavage (Fig. 2A) and trypan blue-positive cells (Fig. 2C); and *iii*) reduced the cell proliferation (Fig. 2C), inducing G_2_/M phase cell cycle arrest (Fig. 2D). Overall, the results demonstrated that the antitumour effects induced by AT9283 recapitulated the MKK3 depletion effects [6–8, 11], suggesting that AT9283 is an MKDMA compound in tested CRC lines.

### The AT9283 mimicks MKK3-depletion effects mainly inhibiting the AURKA functions in CRC lines

**Fig. 3:**
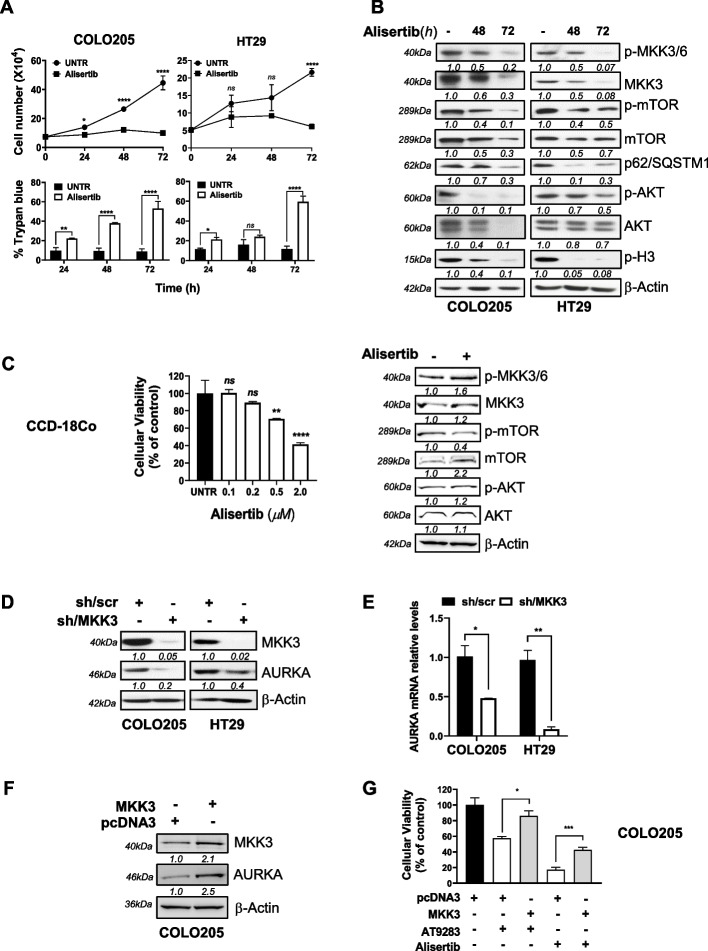
AT9283 triggers anti-tumor effects mainly inhibiting AURKA activity in CRC. **A** Alisertib IC_50_ effects on COLO205 (65 nM) and HT29 (100 nM) cells proliferation and survival. Representative results of three independent experiments performed in technical triplicates are reported as means ± SDs. Significance was assessed with ordinary one-way ANOVA followed by Dunnett’s post hoc multiple comparisons test: ns, not significant; *p < 0.05; **p < 0.01; ****p < 0.0001. **B** Protein lysates, from 48 and 72 h alisertib IC_50_ pre-treated cells, were analysed by WB with the indicated antibodies. More relevant bands from the same filter at the same exposure length are reported; **C** (l*eft panel)*. MTT assays with colonocytes treated 72 h with alisertib at indicated doses. Results, quantified to untreated cells (UNTR), are reported as the mean ± SD. Representative data of three independent experiments in technical triplicates are reported. Significance was assessed with ordinary one-way ANOVA followed by Dunnett’s post hoc multiple comparisons test: ns, not significant; **p < 0.01; ****p < 0.0001; **C** *(right panel)*. Protein lysates from colonocytes pre-treated 72 h with alisertib IC_50_ (2.0 μM), were analysed by WB with the indicated antibodies. More relevant bands from the same filter at the same exposure length are reported.** D** Protein lysates from COLO205 and HT29-sh/scr and -sh/MKK3 sublines pre-treated 96 h with DOX (1 μg/mL) were analysed by WB with the indicated antibodies. More relevant bands from the same filter at the same exposure length are reported. **E** Cells were treated as in **D** and RNAs analysed by qPCR. Data were normalized to GAPDH, quantified with respect to sh/scr set to 1.0, and reported as the mean ± S.D. Representative results of three independent experiments (biological replicates) are reported. Significance was assessed with unpaired Student’s t test: *p < 0.05; **p < 0.01; **F** Protein lysates from transiently transfected (48 h) COLO205-empty (pcDNA3) or HA-tagged MKK3- (pDNA3HA-MKK3) were analysed by WB with the indicated antibodies or (**G**) treated with AT9283 (15 nM) or alisertib (60 nM). Effects on cell proliferation assessed 72 h later by MTT. The results are reported as the mean ± SD of three independent experiments with similar results. Significance was assessed with two-tailed unpaired Student’s t tests: *p < 0.05, ***p < 0.001

Recent OncoPPi network analyses revealed that MKK3, but not its close homologue MKK6, interacts with multiple proteins from various signaling pathways including the Aurora kinase A (AURKA [11]. Interestingly, either AT9283 treatment or MKK3 depletion induced G_2_/M phase cell cycle arrest [6] (Fig. 2D) and stalled mitotic spindle formation (Suppl. Fig. 4C). Hence, we explored whether AT9283 may inhibit MKK3 functions by directly targeting the Aurora kinases activity in CRC. Primary investigations with selective inhibitors showed that alisertib (AURKA) reduced survival in both the COLO205 and HT29 lines (Suppl. Fig. 5A), whereas barasertib (AURKB) impacted on survival only in COLO205 cells (Suppl. Fig. 5B) without perturbing the MKK3 signalling in both CRC line (Suppl. Fig. 5B). By contrast, alisertib IC_50_, albeit to a minor extent when compared to MKK3 depletion [6–8, 11] or AT9283 treatments (Fig. 1D), inhibited cell proliferation in both the COLO205 and HT29 lines (Fig. 3A); induced cell death (Fig. 3A) and autophagy (Fig. 3B); and abrogated the MKK3 and PI3K/AKT signalling pathways (Fig. 3B). The reduced phospho-histone H3(pH3) guaranteed the effectiveness of the AURKA inhibition obtained in the experimental conditions adopted (Fig. 3B). Interestingly, alisertib at doses that trigger consistent anti-tumoral effects in CRC lines (Suppl. Fig. 5A) did not affect the cell viability of primary colonocytes (CCD-18Co) and the MKK3 signalling resulted not affected upon challenging with relative IC_50_ (2.0 µM) (Fig. 3C). These results suggested the relevance of the MKK3/AURKA crosstalk in sustainingCRC malignancy. To deeper understand the relationships between MKK3 and AURKA in CRC cells, we addressed loss- and gain-of-function assays. Efficient MKK3 depletion consistently dropped AURKA at both protein (Fig. 3D) and mRNA (Fig. 3E) levels in both COLO205 and HT29 lines. Similarly, the efficient AURKA knockdown decreased the phospho- and total MKK3 protein levels (Suppl. Fig. 6A) reducing cell viability in both tested CRC lines (Suppl. Fig. 6B). By contrast, ectopically expressed MKK3 increased the AURKA protein levels (Fig. 3F) and rescued the anti-proliferative effects triggered by either AT9283 or alisertib in COLO205 cells (Fig. 3G). Lastly, co-treatment with the proteasome inhibitor (MG132) restored the effects induced by AT9283 or alisertib on MKK3 protein levels (Suppl. Fig. 6C) and promoted cell survival (Suppl. Fig. 6D). Results suggested that both drugs, through the AURKA inhibition, induced MKK3 protein degradation hampering the CRC malignancy in tested lines, and revealed a novel mutual dependency between the AURKA and MKK3 proteins, as activated MKK3 sustaining the AURKA gene expression enforce its own protein stability and activation, supporting CRC malignancy. Overall results suggested AT9283 as MKDMA compound through the AURKA inhibition in CRC cells.

### AT9283 inhibits MKK3/AURKA nuclear co-localization in CRC lines

**Fig. 4:**
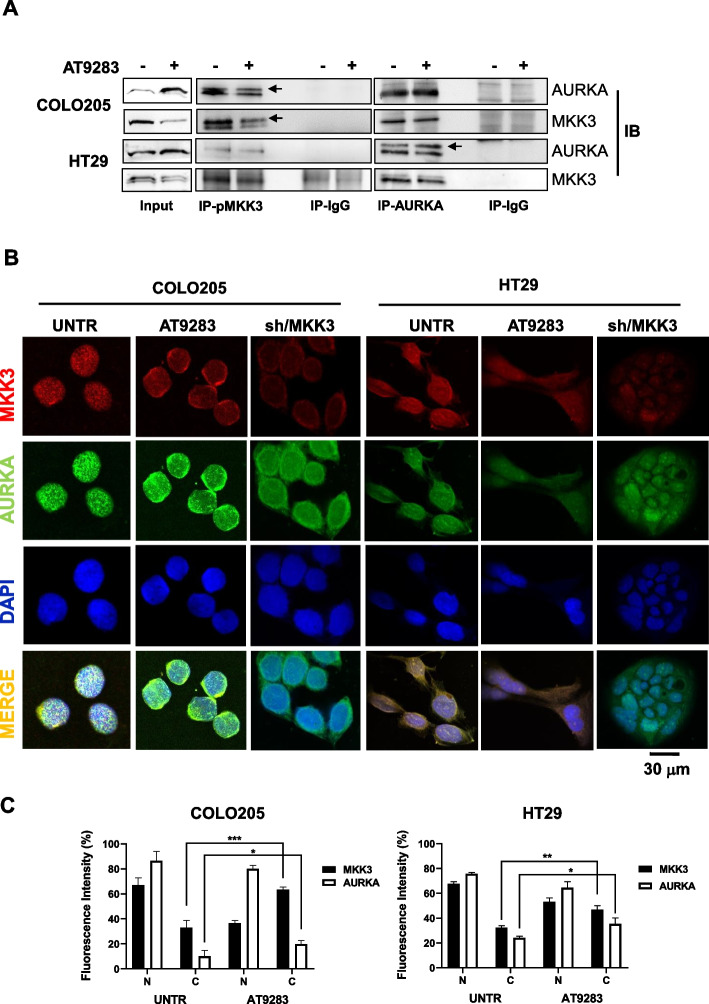
AT9283 abrogates MKK3/AURKA nuclear co-localization in CRC cells.** A** Protein lysates from COLO205 and HT29 cells, left untreated or treated for 72 h with AT9283 at the IC_50_, were immune-precipitated either with the anti-pMKK3 or anti-AURKA antibody, and immuno-complexes were revealed by western blot analysis with the indicated antibodies. More relevant bands from the same filter at the same exposure length are reported; **B** Cells were treated or left untreated, and immunofluorescence was performed with the indicated antibodies. Immunostained cells were analysed at 40X magnification under a confocal microscope. Representative data from three independent experiments with similar results are reported; **C** Quantifications of the intensities of each fluorophore in the nucleus (N) and cytoplasm (C) were performed with ImageJ softwar. Results are reported as the mean ± S.D. Significance was assessed with two-tailed unpaired Student’s t tests: *p < 0.05, ***p < 0.001, ****p < 0.0001. UNTR = untreated

To investigate the AT9283 triggered effects on the MKK3/AURKA crosstalk, co-immunoprecipitation (Co-IP) assays were performed with COLO205 and HT29 cells upon treatments. Results demonstrated that, also in tested CRC cells, MKK3 interacts and complexes to AURKA (Fig. 4A), however the MKK3/AURKA protein–protein interaction was not perturbed by AT9283 treatments in both tested CRC line (Fig. 4A). We next questioned whether MKK3 might co-localize with AURKA and whether the AT9283 treatments might affect the MKK3/AURKA protein co-localization. Co-immunofluorescence staining revealed that MKK3 co-localizes with AURKA in the nucleus and cytoplasmic regions in both COLO205 and HT29 cells (Fig. 4B, C) and, according to Co-IP results, the AT9283 treatments did not abrogate the co-localization but shifted the protein–protein complex to the perinuclear/cytoplasmic (Fig. 4B, C). Interestingly, the MKK3 knockdown hampered the AURKA nuclear localization (Fig. 4B), suggesting that MKK3 may play relevant key roles in regulating AURKA functions in CRC cells. Therefore, the results demonstrate that AT9283 treatments, likely the MKK3 depletion, inhibit the AURKA nuclear localization, in the tested lines, suggesting AT9283 as MKDMA compound in CRC.

### AT9283 abolishes MKK3-induced cell motility in CRC  lines

**Fig. 5:**
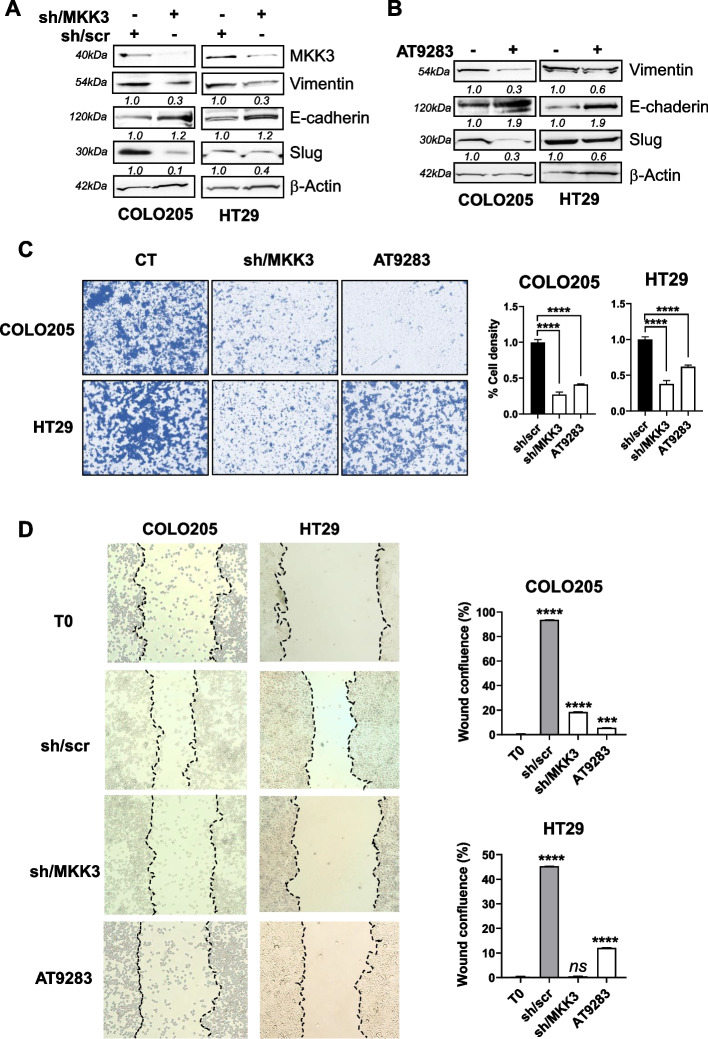
AT9283 mimics the MKK3 depletion effects hampering CRC cell motility.** A**, **B** COLO205 and HT29-sh/scr, -sh/MKK3 sublines were treated for 120 h with DOX (1 μg/mL) (**A**), whereas parental cells were treated for 72 h with AT9283 at the IC_50_ (**B**), and protein lysates were analysed via western blotting with the indicated antibodies. More relevant bands from the same filter at the same exposure length are reported; **C** (left panels). COLO205 and HT29-sh/scr-sh/MKK3 sublines pre-treated for 96 h with DOX (1 μg/mL) and parental cells left untreated or pre-treated for 72 h with AT9283 at the IC_50_ were plated on cell inserts and stained with crystal violet 24 h later. Images were acquired with an optical microscope at 20X magnification. CT, relative control (untreated or sh/scr). Representative images of three independent experiments are shown. **C** (right panels). Quantification of the acquired images of the stained cells was performed with ImageJ software. The representative results of three independent experiments, quantified with respect to relative controls (set to 1.0), are reported as the mean ± SD. Significance was assessed with ordinary one-way ANOVA followed by Dunnett’s post hoc multiple comparisons test: ****p < 0.0001. **D** Wounds were generated at 80% confluence from COLO205- and HT29-sh/scr and -sh/MKK3 sublines pre-treated for 96 h with DOX (1 μg/mL) and from parental cells left untreated or pre-treated for 72 h with AT9283 at the IC_50_. Twenty-four hours later, the wounds were analysed under an optical microscope (20X magnification). Representative images of three independent experiments are shown (left panels). Wounds were quantified with ImageJ software on acquired images and the percentage of confluent wounds was estimated. The representative results of three independent experiments are reported as the mean ± SD (right panel). Significance was assessed with ordinary one-way ANOVA followed by Dunnett’s post hoc multiple comparisons test: ns, not significant; ***p < 0.001; ****p < 0.0001

Invasion and metastasis, ‘hallmarks of cancer’ [40], are the main causes of cancer-related mortality. We previously demonstrated that MKK3 sustains CRC cell-motility as its depletion significantly inhibited migration with HT29 cells [11]. To investigate whether AT9283 may affect MKK3 oncogenic functions abrogating CRC invasiveness, we first assessed the role of MKK3 in modulating the expression of well-known markers of the epithelial-mesenchymal transition (EMT). In both the COLO205 and HT29 lines, the MKK3 depletion raised the epithelial marker E-cadherin (Fig. 5A), while decreased the mesenchymal markers as vimentin, Slug, Snail, Zeb1 and Twist1 (Fig. 5A, suppl. Fig. 7). Moreover, the MKK3 knockdown (KD) reduces cell invasion (Fig. 5C) and cell migration (Fig. 5D) when assessed respectively by Boyden chamber and wound-healing assays in both CRC lines. Parallel investigations demonstrated that AT9283 treatments recapitulated the MKK3 depletion effects modulating similarly the epithelia and mesenchymal markers (Fig. 5B, suppl. Fig. 7), as well as hampering cell invasion (Fig. 5C) and migration (Fig. 5D) with both CRC lines. Overall, these results suggested AT9283 as MKDMA drug in CRC cells.

### AT9283 mimicks MKK3-depletion effects in pre-clinical models and CRC patients derived organoids

**Fig. 6:**
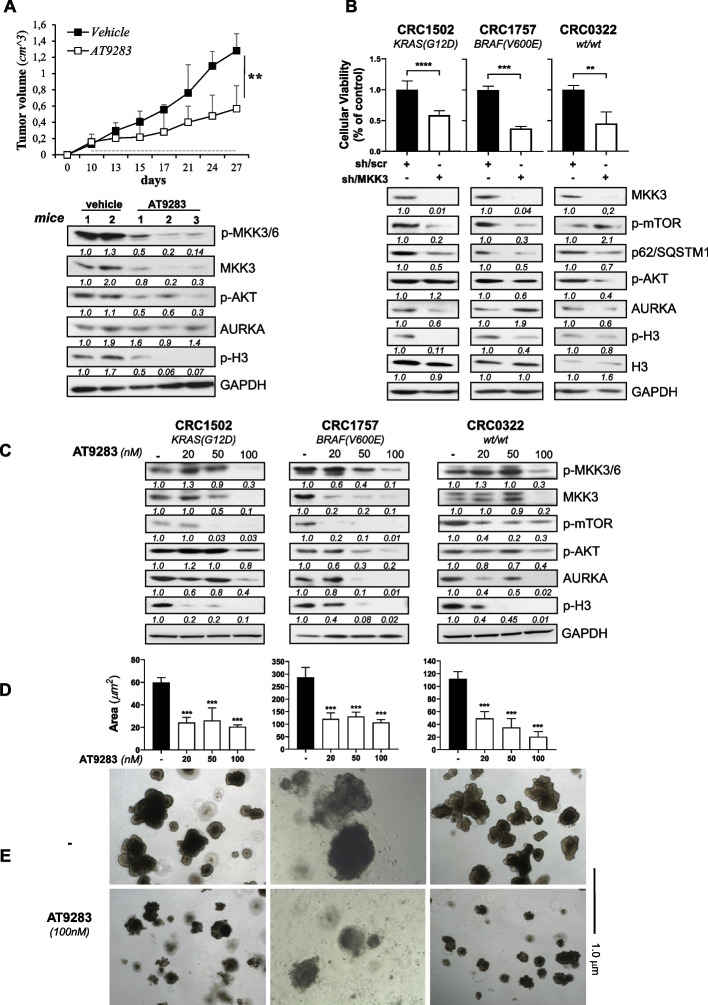
AT9283 recapitulated the effects of MKK3 depletion in preclinical models and in CRC patient-derived organoids.** A** COLO205 xenografted cells (6 mice/group) were treated daily with either AT9283 (15 mg/kg) or vehicle solution. Upper panel: Tumour growth effects are reported as the means ± S.D. Significance was analysed using two-way ANOVA: **p < 0.01. Lower panel: Protein lysates from explanted tumors were analysed by WB with the indicated antibodies. More relevant bands from the same filter at the same exposure length are reported. **B** Engineered -sh/scr, and -sh/MKK3 PDOs were pre-treated 144 h with DOX (1 μg/mL) and effects on cell proliferation assessed by MTT assay (upper panels). Results were quantified with respect to the control set to 1.0 and reported as the mean ± SD. Representative results of three independent experiments are reported. Significance was assessed with two-tailed unpaired Student’s t test: ns, not significant; **p < 0.01; ***p < 0.001, ****p < 0.0001. Lower panels. Protein lysates from engineered sh/scr and sh/MKK3 PDOs, pre-treated 144 h with DOX (1 μg/mL), were analysed by WB with indicated antibodies. More relevant bands from the same filter at the same exposure length are reported. **C** Protein lysates from PDOs pre-treated 144 h with AT9283, were analysed by WB with the indicated antibodies. More relevant bands from the same filter at the same exposure length are reported. **D**, **E** PDOs pre-treated as in **C**, were analysed at microscope (4X magnification) and relative area (μm^2^) quantified with ImageJ software on collected images. The representative results of three independent experiments are reported as the mean ± SD. Significance was assessed with ordinary one-way ANOVA followed by Dunnett’s post hoc multiple comparisons test: ***p < 0.001; **E** Representative images of PDOs left untreated or treated with AT9283 at the highest dosage tested

To validate AT9283 as MKDMA compound with pre-clinical models, mice bearing established COLO205 xenografts (2.0 cm^3^, tumor volume) were randomly selected and treated either with AT9283 (15 mg/kg) or vehicle solution (control) [32–35]. The in vivo AT9283 treatments, similarly to the MKK3 depletion [11], reduced significantly the COLO205 tumor growth when compared with the control group (Fig. 6A: *upper panel*). Western blot analysis of excised tumors revealed that, likely the in vitro data, AT9283 in vivo reduced: *i*) phospho- and total-MKK3 protein levels; *ii*) AURKA activity, assessed by pH3 levels; *iii*) PI3K/AKT signalling pathway reducing the p-AKT levels (Fig. 6A: *lower panel*). To further validated AT9283 as MKDMA drug we selected a subset of CRC patient-derived organoids (PDOs) bearing different mutational backgrounds: CRC1502 (CRC1502LMO0A), CRC1757 (CRC1757LMO0A), and CRC0322 (CRC0322LMO0A) (Table 1). First, we explored the sensitivity to MKK3 knockdown and selected CRC-PDOs were engineered with inducible shRNA system to generate sh/scr and sh/MKK3 derivatives. According to results with CRC lines, also with selected CRC-PDOs, the DOX-induced MKK3 depletion (Fig. 6B): *i)* inhibited significantly the proliferation and survival in all the tested CRC-PDOs (Fig. 6B, *upper panels*); ii) induced autophagy; *iii*) inhibited the AKT/mTOR and the AURKA signalling pathways (Fig. 6B, *lower panels*). Next, to corroborate AT9283 as MKDMA compound also with highly relevant CRC models, selected PDOs were challenged with escalating doses of drug and the effects assessed. According with the in vitro and in vivo results, AT9283 dose-dependently decreased the phospho- and total-MKK3 protein levels, the AKT/mTOR, and AURKA signalling pathways, and inducing sustained autophagy (Fig. 6C). Moreover, AT9283 reduced significantly and dose-dependently the size of treated tumoroids (area μm2), when compared to controls (Fig. 6D, Suppl. Fig. 8), reflecting the inhibitory effects on proliferation and survival (Fig. 6D, E, and Suppl. Fig. 8). Finally, similarly to results with CRC lines (Fig. 1E), AT9283 modulated the MKK3-dependent gene signature also in tested CRC-PDOs (Suppl. Fig. 9). Overall, these results validated AT9283 as MKDMA drug also in pre-clinical and highly relevant CRC models.

## Discussion

Advanced-stage and metastatic CRC is still a fatal disease, making the identification of novel molecular targets imperative for the development of more selective and efficient therapeutic strategies.

Previous studies have suggested MKK3 as an attractive and promising therapeutic target, prompting us to further investigate to provide the basis for the development of new strategies for the treatment of patients with advanced and metastatic CRC [6–8, 11, 12]. MKK3-specific inhibitors are lacking, and some recently developed [41] are affected by poor specificity and still need to be validated in preclinical settings. In this study, we investigated whether a drug repurposing approach based on transcriptome data may identify FDA-approved drugs with inhibitory activity of the MKK3 signalling and thus mimicking the well-known biologic effects triggered by the inducible RNAi mediated silencing of MKK3 seen in CRC cells. Gene expression profiling with HT29 cells revealed 1799 DEGs (590 upregulated and 1209 downregulated genes) upon MKK3 depletion when compared to the control (sh/scr) (Fig. 1A), highlighting the relevance of MKK3 in modulating gene expression in tested CRC cells. TCGA-COADREAD analyses identified two relevant signatures MKK3 dependent, consisting of 52 unique downregulated genes with positive prognostic value (Suppl. Table 1) and 16 upregulated genes with negative prognostic valu**e** (Suppl. Table 2). The querying of identified prognostic gene signatures in the NIH-LINCS database identified AT9283 among the top-scoring compounds, which has been selected for further investigations to corroborate as MKMDA drug. Exploitations with a panel of CRC lines and PDOs bearing different mutational setting and primary colonocytes demonstrated that interestingly the AT9283 treatments impacted on MKK3 protein levels reducing both phosphorylated and total protein (Fig. 1C) in all tested CRC lines. Deeper investigations revealed that AT9283 impacted on MKK3 at different levels by inducing protein degradation via the proteasome (Suppl. Fig. 6C, D) and reducing the MKK3 mRNA levels (suppl. Fig. 4B). Moreover, AT9283 induced anti-tumor effects, recapitulating the MKK3 depletion effects, in all tested CRC cell lines, as: *i*) induced autophagy (Fig. 2A, B) and cell death (Fig. 2A, C, D); *ii*) induced G2/M phase cell cycle arrest (Fig. 2C, D); *iii*) reduced cell migration and invasion in vitro (Fig. 5); *iv*) reduced tumour growth in pre-clinical models (Fig. 6A). To further validate AT9283 as MKDMA drug, analyses at transcriptional levels demonstrated that AT9283 treatments modulated at similar extent the identified MKK3-dependent gene signatures with both tested CRC lines (Fig. 1E). Noteworthy, AT9283 treatments, like MKK3 depletion, resulted well-tolerated when tested in primary colonocytes (Fig. 1C, D). Corroborative investigations with subsets of CRC-PDOs revealed, that independently from their mutational setting, all tested PDOs responded at similar extent to the MKK3 depletion (Fig. 6B), therefore suggesting that the MKK3 targeting might cover a wider therapeutic opportunity in CRC. These results appear in contrast with those achieved with CRC lines, which revealed the BRAF-mutated subtype as the most dependent to MKK3-driven pro-survival signalling [8, 11]. Therefore, further investigations are needed to better evaluate the reliability of different experimental models in the CRC disease and therapeutic outcomes. The AT9283 treatments abrogated dose-dependently the MKK3 protein levels and inhibited the AKT/mTOR and AURKA signalling in all tested PDOs (Fig. 6C), which correlated with significant reduction in tumoroids size (Fig. 6D, E). According to results in CRC lines, AT9283 treatments modulated the MKK3-dependent gene signature in all tested PDOs (Suppl. Fig. 9). Overall results corroborated AT9283 as a MKDMA drug also with clinically relevant CRC-PDOs. At molecular levels, we demonstrated that AT9283 treatments abrogated the MKK3 protein levels mainly through the targeting of the AURKA signalling, shedding lights on the relevance of MKK3/AURKA crosstalk in sustaining CRC malignancy. Indeed, activated MKK3 supoorts AURKA gene expression (Fig. 3D, E), thus promoting its own protein stability and activity (Suppl. Fig. 6), hence supporting CRC cell proliferation, survival (Fig. 3G, Suppl. Fig. 6) and motility (Fig. 5). Interestingly, co-immunofluorescence staining revealed novel MKK3 roles in promoting AURKA functions, since MKK3 co-localized with AURKA both in the cytoplasmic and nuclear regions, and the MKK3-knockdown affected the AURKA nuclear localization (Fig. 4B), wheras the AT9283 treatments shifted the protein complex in cytoplasmic / perinuclear regions (Fig. 4B) without altering the protein–protein interaction in tested CRC lines (Fig. 4A). Overall, albeit more in-depth investigations are needed to fully understand the importance of the proposed regulatory network in sustaining the CRC malignancy, that are beyond the interest of this work, these results are suggesting that MKK3 may play key roles in regulating AURKA functions in tested CRC cells.

## Conclusions

In summary, in the current study, we provide the proof of concept that drug repurposing approach based on transcriptome data is feasible toll to identify FDA-approved drugs to target MKK3 signalling. Our data demonstrated that AT9283 is indeed is a promising MKDMA drug for the development of novel therapeutic strategies to target MKK3 oncogenic functions in late-stage and metastatic CRC disease. Since, the anti-tumoral effects triggered by AT9283 treatment recapitulated manly the MKK3 depletion effects in all tested CRC models. Our data demonstrated that AT9283 abrogated MKK3 functions mainly through AURKA inhibition highlighting the relevance of the MKK3/AURKA crosstalk in sustaining CRC malignancy, and provide novel insights on the regulatory network between these two proteins in CRC. Interestingly the well-tolerance and the absence of effects on MKK3 signalling of either AT9283 or alisertib when tested in primary colonocytes (CCD-18Co) suggested the relevance of MKK3/AURKA crosstalk only in cancer cell context, prompting to suggest a novel vulnerability to be targeted for the development of novel therapeutic strategies to manage late-stage and metastatic CRC patients.

### Supplementary Information

Supplementary Material 1.

Supplementary Material 2

## Data Availability

The datasets used and/or analysed during the current study are available from the corresponding author upon reasonable request.

## Abbreviations

5-FU: 5-Fluorouracil
Abl: Abelson tyrosine-protein kinase
ATCC: American Type Culture Collection
AURKA: Aurora kinase A
AURKB: Aurora kinase B
BME: Basement membrane extracts
CAPOX/XELOX: Capecitabine and oxaliplatin
COADREAD: Colorectal adenocarcinoma
CRC: Colorectal cancer
DEG: Differentially expressed gene
DFS: Disease-free survival
DMEM: Dulbecco's modified Eagle's medium
DMSO: Dimethyl Sulphoxide
DOX: Doxycycline
ECACC: European Collection of Authenticated Cell Cultures
ECL: Enhanced chemiluminescence
EMT: Epithelial-mesenchymal transition
FBS: Fetal Bovine Serum
FDA: Food and Drug Administration
FLT3: Fms Related Receptor Tyrosine Kinase 3
FOLFIRI: Leucovorin, 5-FU, and irinotecan
FOLFOX: Leucovorin, 5-FU, and oxaliplatin
GAPDH: Glyceraldehyde-3-phosphate dehydrogenase
GUSB: Glucuronidase beta
H3: Histone H3
HRP: Horseradish peroxidase
IHC: Immunohistochemistry
JAK: Janus kinase
LC3: Light chain 3
LINCS: Library of Integrated Network-Based Cellular Signatures
MAPK: Mitogen activated protein kinase
MKDMA: MKK3 knockdown mimicking activity
MKK3: Mitogen activated protein kinase kinase 3
MOI: Multiplicity of infection
MTT: 3-(4,5-Dimethylthiazol-2-yl)-2,5-dipheniltetrazolium bromide
Onco-PPI: Protein‒protein interaction network analyses of cancer-associated genes
OS: Overall survival
PBS: Phosphate-buffered saline
PCR: Polymerase chain reaction
PDO: Patient-derived organoid
PEG: Polyethylene glycol
RIPA: Radioimmunoprecipitation assay
RNU2: U2 Small Nuclear 1
RMA: Robust Multiple-array Average
RPL19: Ribosomal protein L19
RPMI: Roswell Park Memorial Institute
RT: Room temperature
SQSTM1: Sequestosome-1
SCR: Scramble
TCGA: The Cancer Genome Atlas
TET: Tetracycline
TMA: Tissue Microarrays
WB: Western blotting
