# Supplementary material for: Repurposed AT9283 triggers anti-tumoral effects by targeting MKK3 oncogenic functions in Colorectal Cancer
Source: J Exp Clin Cancer Res. 2024 Aug 20;43:234. doi: 10.1186/s13046-024-03150-4 (PMC11334304; doi:10.1186/s13046-024-03150-4)
Supplement: Supplementary file 1 — Supplementary Material 1. [file 13046_2024_3150_MOESM1_ESM.docx]

**REPURPOSED AT9283 TRIGGER ANTI-TUMORAL EFFECTS BY TARGETING MKK3 ONCOGENIC FUNCTIONS IN COLORECTAL CANCER**

Valentina Piastra^1,2^, Federica Ganci^1^, Andrea Sacconi^1^, Angelina Pranteda^1,2^, Matteo Allegretti^1^, Roberta Bernardini^3^, Martina Serra^1,3^, Barbara Lupo^4.5^, Emanuela Dell’Aquila^6^, Gianluigi Ferretti^7^, Edoardo Pescarmona^8^, Armando Bartolazzi^9^, Giovanni Blandino^1^, Livio Trusolino^4,5^ and Gianluca Bossi^1,^*.

1. Translational Oncology Research Unit, Department of Diagnostic Research and Technological Innovation, IRCCS - Regina Elena National Cancer Institute, Rome, Italy;
2. Department of Science, Roma Tre University, Rome, Italy;
3. Interdepartmental Centre for Comparative Medicine, Alternative Techniques and Aquaculture (CIMETA), University of Tor Vergata, Rome, Italy;
4. Candiolo Cancer Institute - FPO IRCCS, Candiolo, Torino, Italy;
5. Department of Oncology, University of Torino, Candiolo, Torino, Italy;
6. Second Division of Medical Oncology, IRCCS - Regina Elena National Cancer Institute, Rome, Italy;
7. First Division of Medical Oncology, IRCCS - Regina Elena National Cancer Institute, Rome, Italy;
8. Department of Pathology, IRCCS - Regina Elena National Cancer Institute, Rome. Italy;
9. Pathology Research Laboratory, Sant'Andrea Hospital, Rome, Italy;

***Corresponding Author.** Gianluca Bossi, Translational Oncology Research Unit, Department of Diagnostic Research and Technological Innovation, IRCCS - Regina Elena National Cancer Institute, Rome, Italy, Via Elio Chianesi 53 - 00144 Rome, Italy.

Phone: +39 06 5266 6995

Fax: +39 06 5266 2740

Email: gianluca.bossi@ifo.it.

**Running title.** AT9283 abolishes MKK3/AURKA crosstalk, reducing CRC malignancy

**SUPPLEMENTARY FIGURES LEGENDS.**

**Supplementary Figure 1. A.** Protein lysates collected at indicate times from HT29-sh/scr and sh/MKK3 sublines treated with DOX (1μg/ml), were analyzed by WB with indicated antibodies. More relevant bands from the same filter at same exposure length are reported; **B.** Protein lysates were collected from HT29-sh/scr and sh/MKK3 sublines treated 60 h with DOX (1μg/ml) in biologic replicates and analyzed by WB with antibodies indicated. More relevant bands from the same filter at same exposure length are reported.

**Supplementary Figure 2.** **A.** Volcano plot of DEGs in sh-MKK3 compared to control sh/scr condition. Most significantly modulated genes are highlighted; **B.** Principal Component Analysis of 1799 DEGs comparing sh-MKK3 and sh-scr RNAs analyzed with Affymetrix gene expression profiling.

**Supplementary Figure 3.** Disease Free Survival (DFS) from TCGA COADREAD generated with the average expression of downregulated (n. 52) (**A**) and upregulated (n. 16) (**B**) genes in sh/MKK3 subline when compared to control (sh/scr). Patients were categorized into high and low signal intensity groups based on positive and negative z-score, respectively. Log-rank test evaluated differences between survival curves. Multivariate Cox Hazard regression model was utilized with adjustments made for T, N, M, stage, and MSI status.

**Supplementary Figure 4. A**. CRC lines were treated with indicated doses of AT9283 and 72 h later effects on cell proliferation assessed by MTT assays. Half-maximal inhibitory concentration (IC_50_) (dashed red line) was estimated for CRC each line through XY plots with GraphPad Prism 8.2. Results were quantified with respect to controls (untreated cells) set to 1.0 and reported as means ± S.D. Representative result of three independent experiments in technical triplicate are shown; **B.** COLO205 and HT29 lines treated with respective AT9283 IC_50_ (15nM and 100nM respectively), and 48 h later total RNAs were analyzed by qPCR with MKK3 and actin (reference gene) specific primer sets. Results were quantified with respect to relative controls (CT) set to 1.0 and reported as means ± S.D. Representative result of three independent experiments in technical triplicate were reported Significance was assessed with unpaired Student’s t-test: *p < 0.05; **C.** α-Tubulin immunofluorescences were performed with HT29-sh/scr and -sh/MKK3 subline pre-treated 96 h with DOX (1μg/ml) and partental HT29 cells untreated or pre-treated 72 h with AT9283 (100nM). α-Tubulin (Red) and DAPI (Blue). Representative images of three independent experiments were reported. CT represents immunofluorescences from untreated and sh/scr cells with similar results.

**Supplementary Figure 5. A.** CRC lines were treated either with Alisertib (*upper panels*) or Barasertib (*lower panels*) at indicated doses, and 72 h later proliferation assessed by MTT assays. Results were quantified with respect to untreated cells set to 1.0 and reported as mean ± SD. Representative result of three independent experiments performed in technical triplicate are reported; **B.** COLO205 and HT29 cells were treated with Barasertib (400nM and 1µM respectively) or left untreated and 72h later protein lysate analysed by WB with indicated antibodies. More relevant bands from the same filter at same exposure length are reported.

**Supplementary data Figure 6. A.** COLO205 and HT29 cells were transiently transfected with control (si/CT) (80nM) or AURKA siRNAs (si/AURKA) (20 or 80nM) and 48 h later protein lysate analysed by WB with indicated antibodies. More relevant bands from the same filter at the same exposure length are reported; **B.** COLO205 and HT29 cells were transiently transfected either with si/CT or si/AURKA (80nM) and 72h later effects on cell proliferation assessed by MTT assays. Results were quantified with respect to controls (100%) and reported as mean ± SD. Representative result of three independent experiments in technical triplicate are reported. Significance was assessed by unpaired Student’s t-test: **p < 0.01; ***p < 0.001; **C.** COLO205 and HT29 cells were treated respectively either with AT9283 (15Nm, 100nM) or Alisertib (65Nm, 300Nm) for 48h and last 4h with MG132 (1µM) or vehicle solution. Protein lysates were analysed by WB with indicated antibodies. More relevant bands from the same filter at the same exposure length are reported. **D.** COLO205 cells were transiently transfected either with si/CT or si/AURKA siRNAs (80nM) and 72h later treated 4h with MG132 (1µM). Effects on cell proliferation were assessed by MTT assays. Results are reported as mean ± SD and quantified with respect to control (si/CT) set to 100%. Representative result of three independent experiments in technical triplicate are shown. Significance was analysed using unpaired Student’s t-test: **p < 0.01.

**Supplementary data Figure 7.** RNAs was isolated from: I) sh/MKK3 and sh/scr sublines treated 144 h with DOX (1 μg/ml); II) AT9283 treated COLO205 (15 nM) and HT29 (100 nM) cells 48 h. Expression of EMT related transcription factors was assessed by qPCR. Results were normalized to GAPDH housekeeping gene and quantified with respect to relative controls (untreated; sh/scr) set to 1.0. Representative results of three independent experiments in technical triplicates are reported. Significance was assessed with an unpaired Student’s t test: *p < 0.05; **p < 0.01; ***p < 0.001; ****p < 0.0001.

**Supplementary data Figure 8. A.** Selected PDOs were left untreated or treated with AT9283 at indicated doses. Treated and untreated PDOs were analysed upon 144h of treatments under microscope (4X magnification) and acquired images analysed with ImageJ software. Representative images from three independent experiments are shown.

**Supplementary data Figure 9.** PDOs were left untreated of treated with AT9283 (100nM), and 96-120 h later total extracted RNAs were analysed by qPCR with primer sets specific to genes indicated. Actin was used as reference gene. Results are quantified with respect to control set to 1.0 and reported as mean ± SD. Representative result of three independent experiments in technical triplicate are shown. Significance was tested with unpaired Student’s t-test: **p < 0.01, ***p < 0.001; ****p < 0.0001.
