# Supplementary material for: Repurposed AT9283 triggers anti-tumoral effects by targeting MKK3 oncogenic functions in Colorectal Cancer
Source: J Exp Clin Cancer Res. 2024 Aug 20;43:234. doi: 10.1186/s13046-024-03150-4 (PMC11334304; doi:10.1186/s13046-024-03150-4)
Supplement: Supplementary file 2 — Supplementary Material 2 [file 13046_2024_3150_MOESM2_ESM.pdf]

A

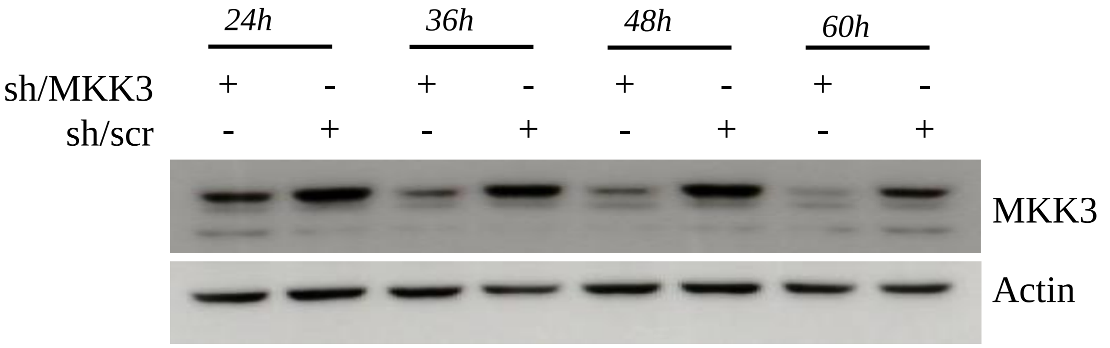
24h
36h
48h
60h
sh/MKK3 + - + - + - + -
sh/scr - + - + - + - +
MKK3
Actin

B

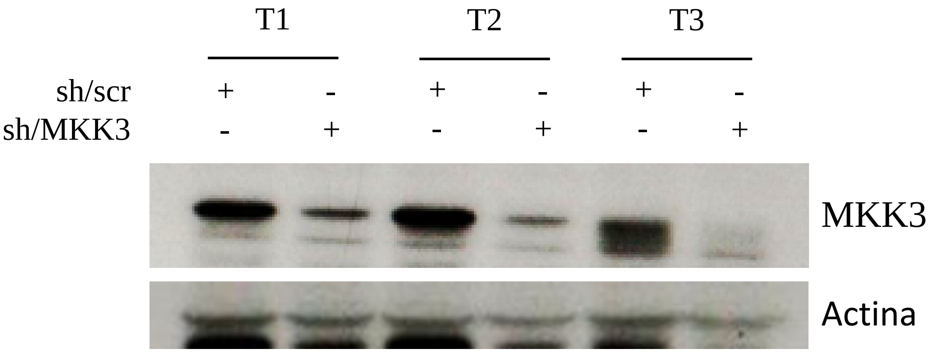
T1
T2
T3
sh/scr + - + - + -
sh/MKK3 - + - + - +
MKK3
Actina

A

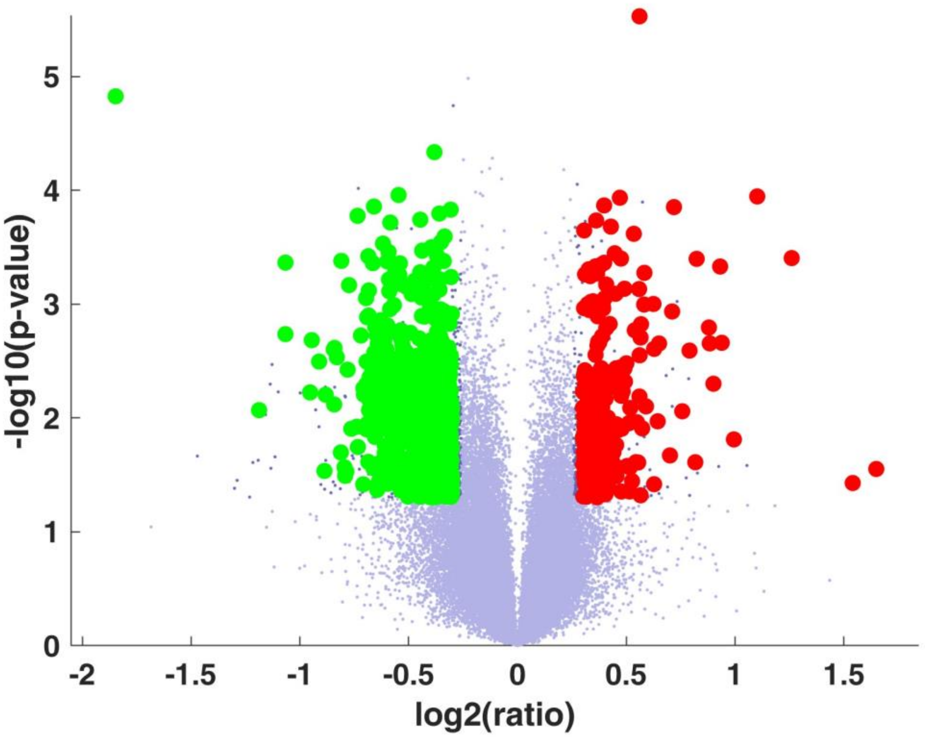
-log10(p-value)
log2(ratio)
0
1
2
3
4
5
-2
-1.5
-1
-0.5
0
0.5
1
1.5

B

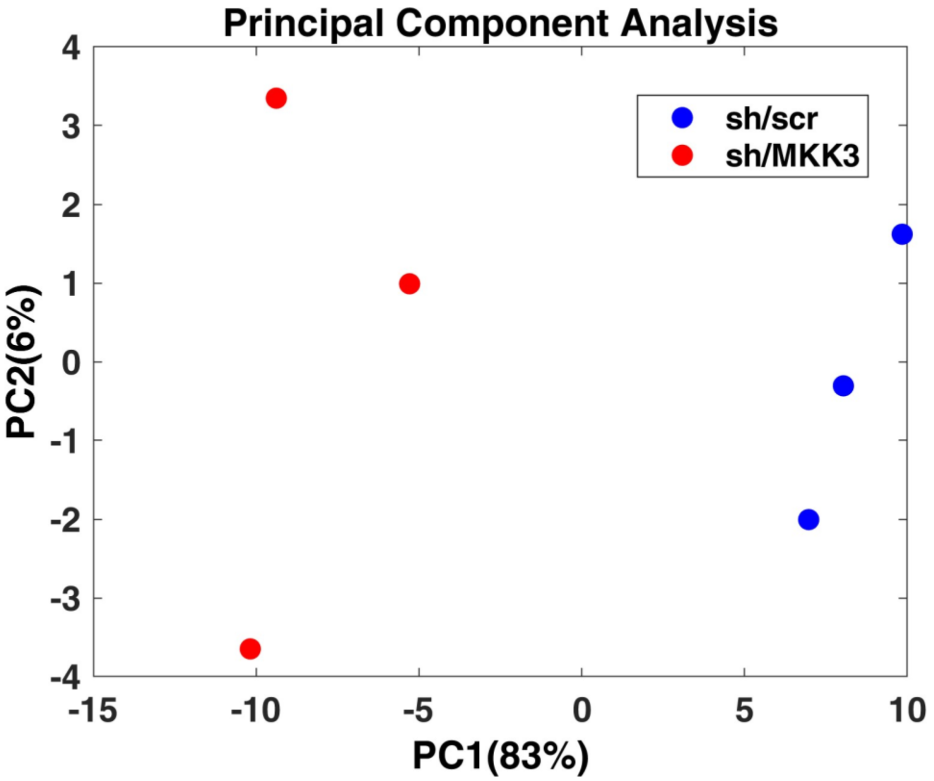
Principal Component Analysis
sh/scr
sh/MKK3
PC2(6%)
PC1(83%)
4
3
2
1
0
-1
-2
-3
-4
-15
-10
-5
0
5
10

A

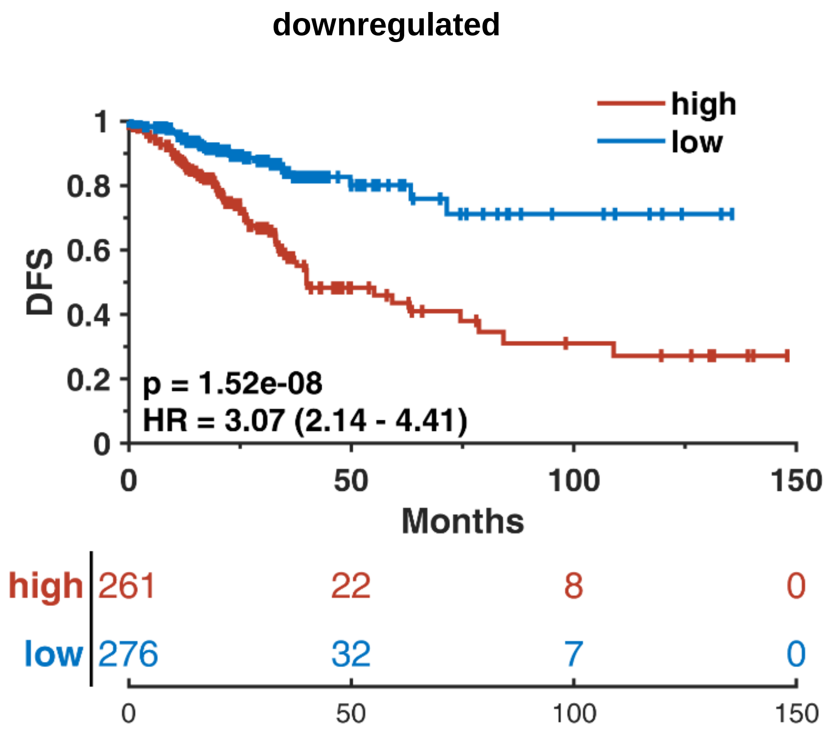
downregulated
high
low
1
0.8
0.6
0.4
0.2
0
DFS
p = 1.52e-08
HR = 3.07 (2.14 - 4.41)
0
50
100
150
Months
high 261 22 8 0
low 276 32 7 0
0
50
100
150

B

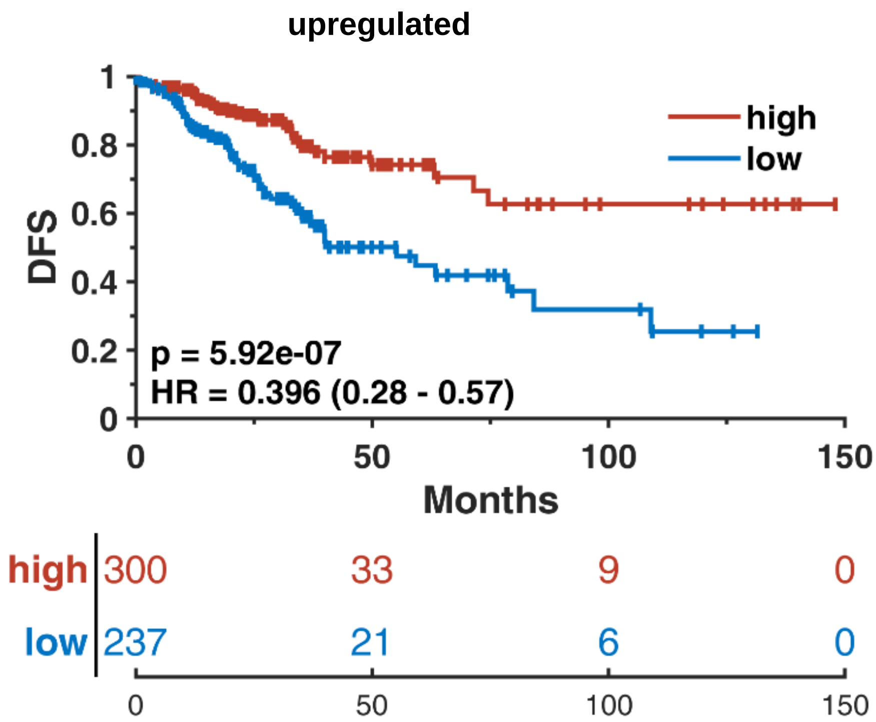
upregulated
high
low
1
0.8
0.6
0.4
0.2
0
DFS
p = 5.92e-07
HR = 0.396 (0.28 - 0.57)
0
50
100
150
Months
high 300 33 9 0
low 237 21 6 0
0
50
100
150

A

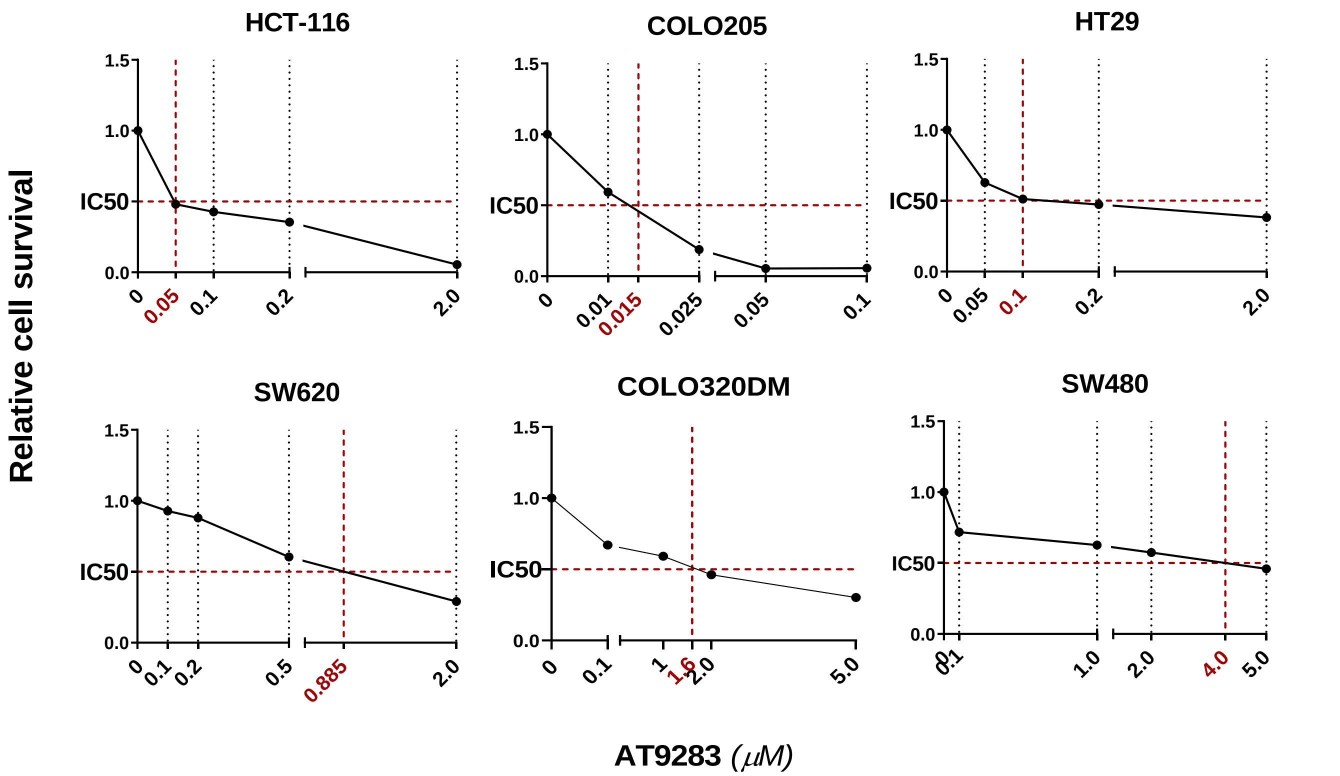
HCT-116
COLO205
HT29
SW620
COLO320DM
SW480
Relative cell survival
IC50
1.5
1.0
0.0
0
0.05
0.1
0.2
2.0
0.01
0.015
0.025
0.05
0.1
0.5
0.885
1
1.6
2.0
5.0
1.0
4.0
AT9283 (μM)

B

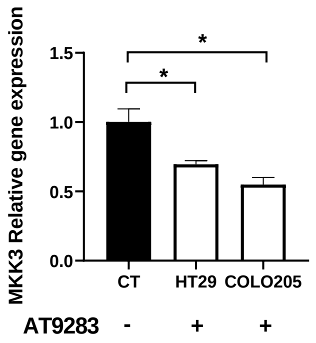
MKK3 Relative gene expression
1.5
1.0
0.5
0.0
*
*
CT
HT29
COLO205
AT9283
-
+
+

C

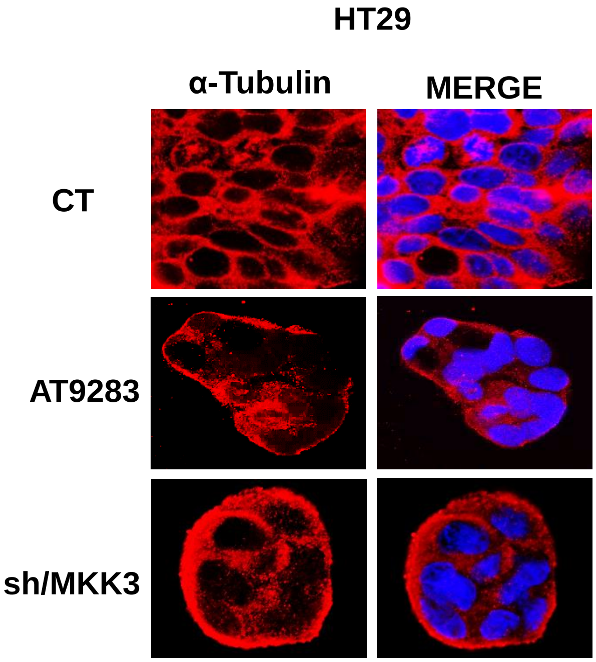
HT29
α-Tubulin
MERGE
CT
AT9283
sh/MKK3

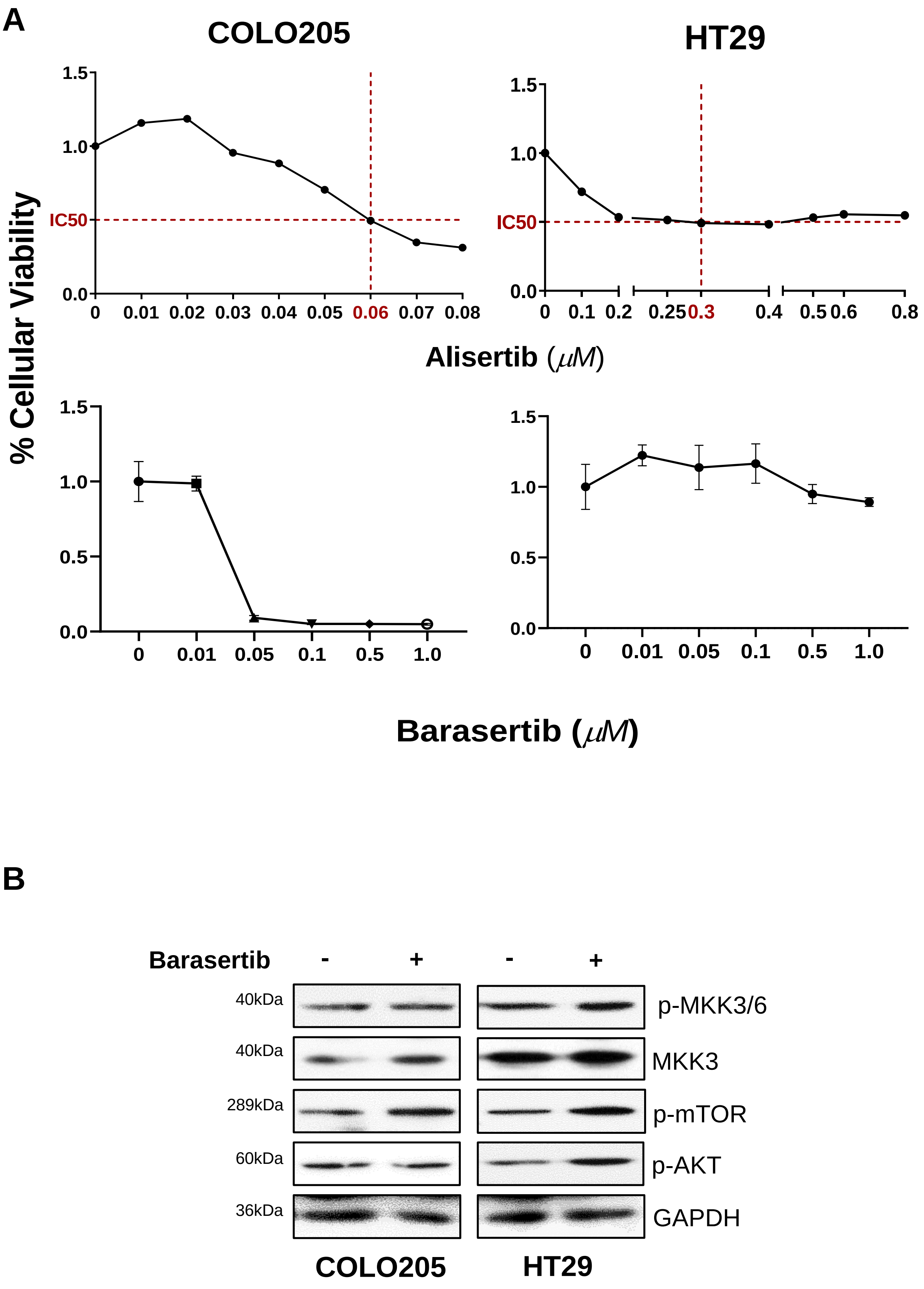
A
COLO205
HT29
% Cellular Viability
1.5
1.0
IC50
0.0
0
0.01
0.02
0.03
0.04
0.05
0.06
0.07
0.08
0.1
0.2
0.25
0.3
0.4
0.5
0.6
0.8
Alisertib (μM)
0.5
0.01
0.05
0.1
0.5
1.0
Barasertib (μM)
B
Barasertib
-
+
40kDa
289kDa
60kDa
36kDa
p-MKK3/6
MKK3
p-mTOR
p-AKT
GAPDH
COLO205
HT29

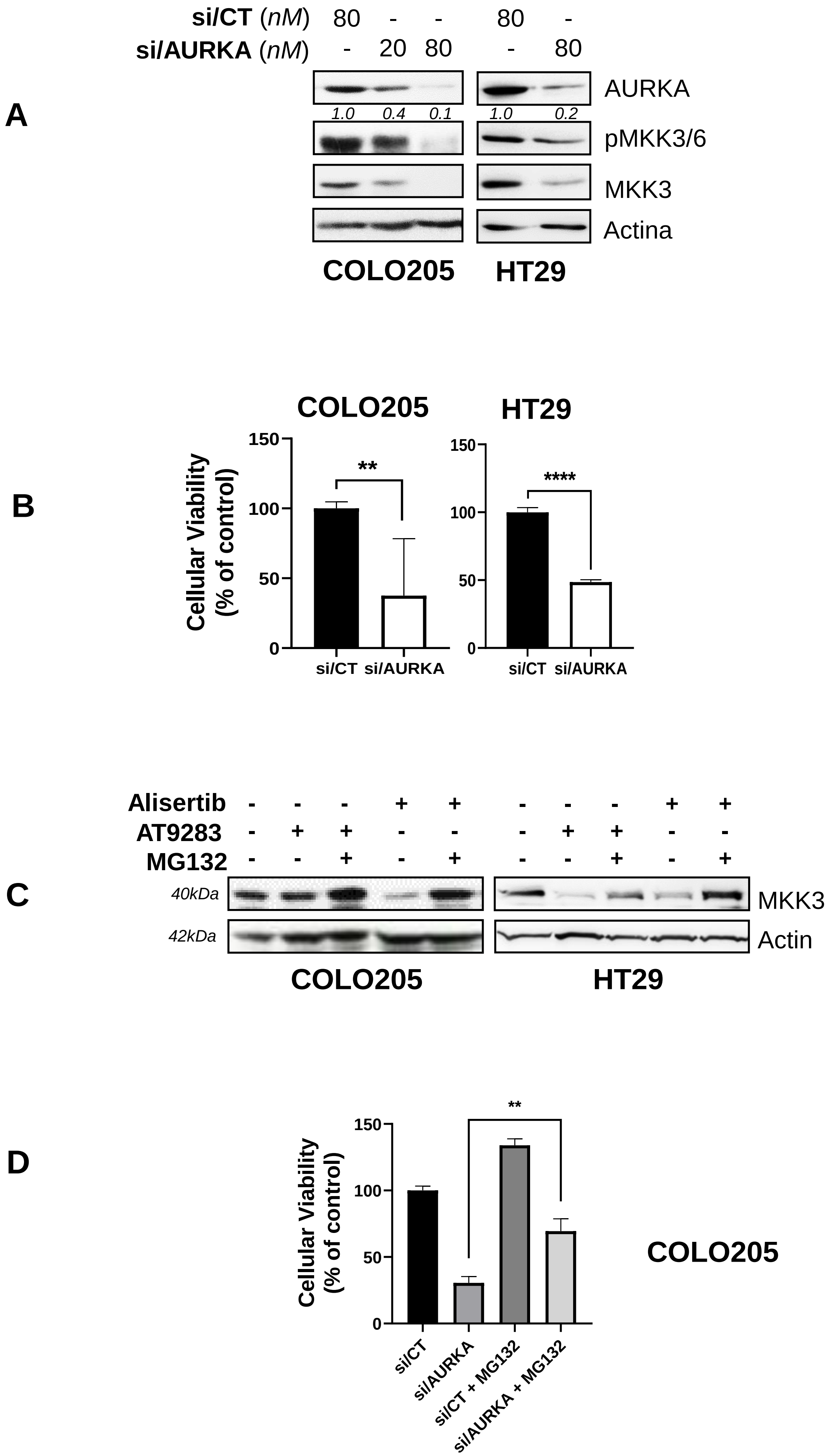
A
si/CT (nM) 80 - - 80 -
si/AURKA (nM) - 20 80 - 80
AURKA
1.0 0.4 0.1 1.0 0.2
pMKK3/6
MKK3
Actina
COLO205
HT29
B
COLO205
HT29
Cellular Viability (% of control)
150
100
50
0
**
****
si/CT
si/AURKA
C
Alisertib - - - + + - - - + +
AT9283 - + + - - - + + - -
MG132 - - + - + - - + - +
40kDa
42kDa
MKK3
Actin
COLO205
HT29
D
Cellular Viability (% of control)
150
100
50
0
**
si/CT
si/AURKA
si/CT + MG132
si/AURKA + MG132
COLO205

COLO205

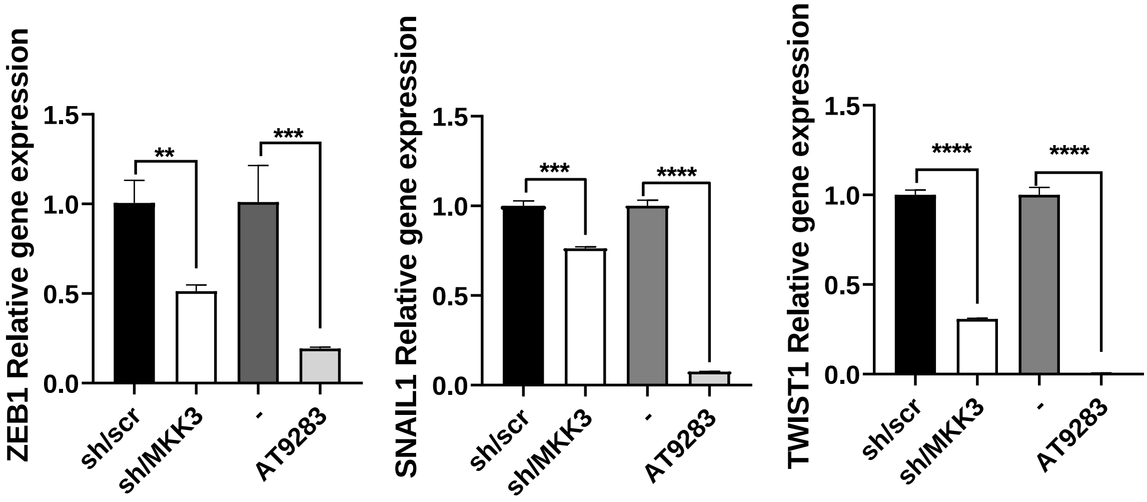

HT29

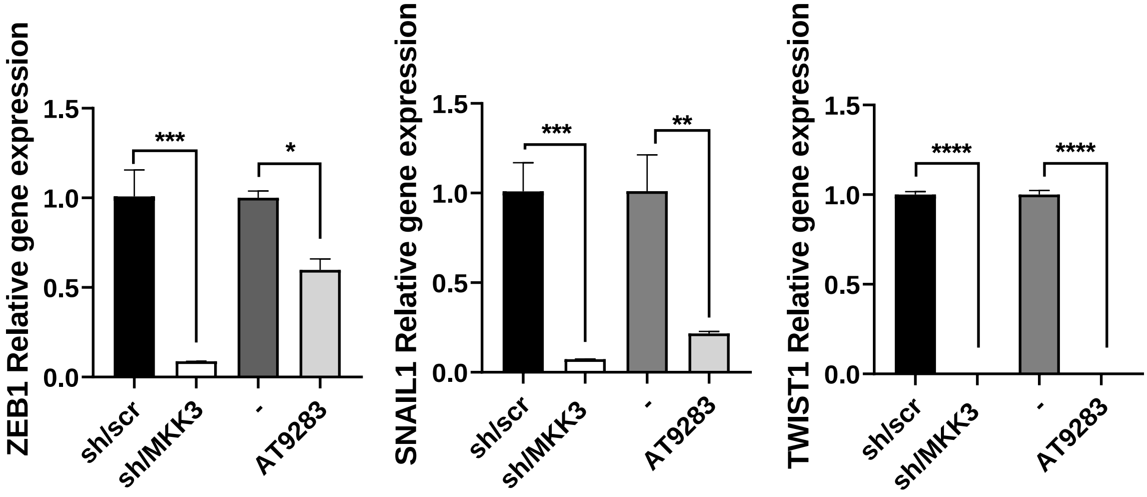

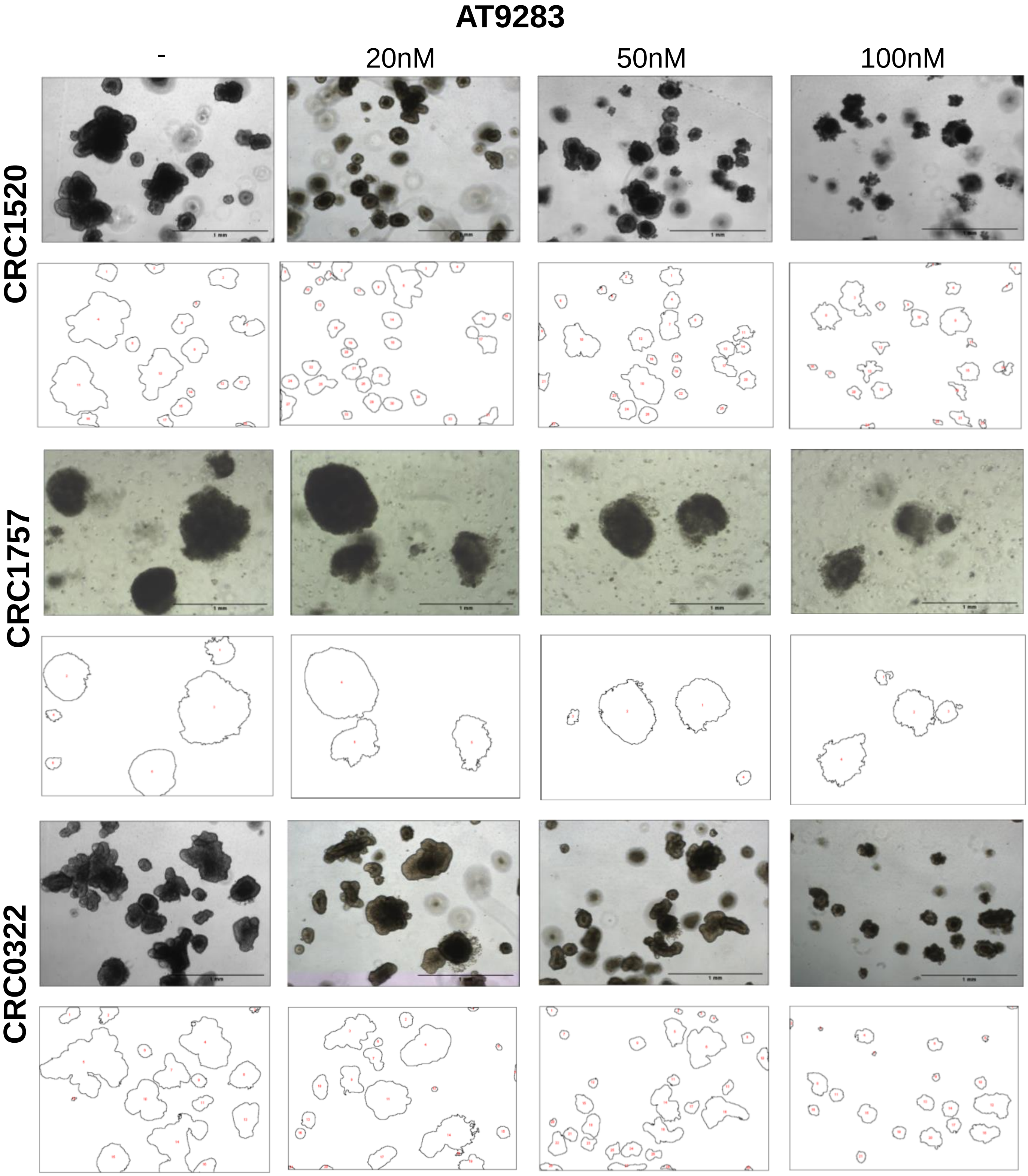
AT9283
-
20nM
50nM
100nM
CRC1520
CRC1757
CRC0322
1 mm

CRC1502
KRAS(G12D)

CRC0322
wt/wt

CRC1757
BRAF(V600E)

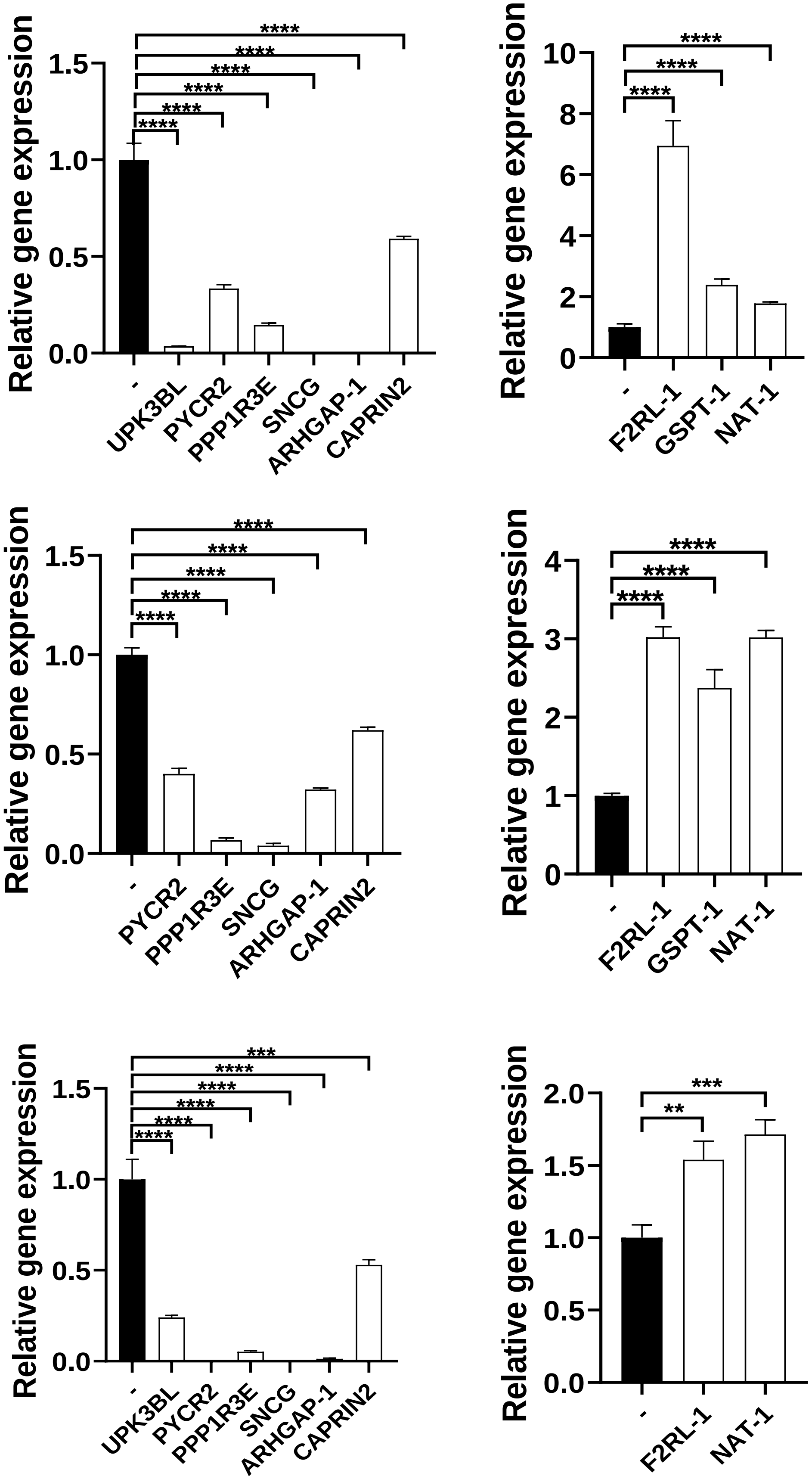

Suppl. Table 1. Downregulated Gene Signature upon MKK3 depletion.

	OS			*DFS*				
Gene	HR cox univ	p	logrank p(zscore)	HR cox univ	p	logrank p(zscore)	permut p	log2FC
UPK3BL**	1,112909845	0,030976	0,017042424	1,210198478	0,000524	1,22635E-05	0,0308425	-0,18679
PYCR2**	1,684170932	0,006765	0,043624663	2,048463286	0,000188	7,3656E-05	0,00975781	-0,20537
CLK2	1,770477711	0,00092	0,051204251	2,102635223	4,37E-05	0,000272737	0,0201504	-0,21974
HOXB8	1,067621979	0,168977	0,652237833	1,30087858	2,27E-05	0,000309823	0,04529823	-0,2654
C16orf52	1,212466617	0,179344	0,109252189	1,564271016	0,00607	0,000684234	0,03667646	-0,28329
FN3KRP	1,139190229	0,515689	0,434458444	1,642724909	0,017969	0,001755137	0,03855043	-0,25411
C1orf116	1,199962867	0,163274	0,399344972	1,322794466	0,046377	0,00474344	0,03582594	-0,41589
CAPRIN2**	1,29565335	0,015267	0,026157403	1,283191271	0,027133	0,005542063	0,01399015	-0,5315
POGZ	1,347544419	0,088171	0,678431369	1,863840487	0,000709	0,006531207	0,00330274	-0,24109
PPP1R3E**	1,382192069	0,003856	0,037010011	1,57182009	0,000138	0,010601898	0,0021504	-0,29638
PGBD2	1,205250611	0,308261	0,472882722	1,893584008	0,000393	0,011511469	0,04455315	-0,23658
ARHGAP23	1,136767361	0,113358	0,550978732	1,33855255	0,00077	0,012286386	0,01617699	-0,21484
FAM122B	1,113730559	0,390533	0,205092703	1,345801635	0,031559	0,013196567	0,02724938	-0,3478
SHC1	1,464774705	0,094777	0,198017079	1,992316932	0,003316	0,013737922	0,03220555	-0,24643
KATNAL1	1,124242815	0,210744	0,639634529	1,291746407	0,011909	0,014670478	0,00041775	-0,33877
PGAP3	1,066505181	0,488416	0,577879941	1,254738834	0,00875	0,016110666	0,03464602	-0,21282
SFTA2	1,067401899	0,089413	0,269446724	1,113714956	0,008305	0,016191487	0,02633218	-0,34227
FARP1	1,037632549	0,686363	0,986177905	1,285840763	0,016906	0,018914653	0,01799706	-0,17662
SNCG**	1,155457716	0,011861	0,005492812	1,129548009	0,054349	0,019549622	0,04122718	-0,32315
CENPJ	0,952527165	0,696578	0,937858486	1,496166561	0,002043	0,020629322	0,04085794	-0,3532
RUNX2	1,099952724	0,202007	0,606030891	1,229717983	0,010872	0,020855976	0,00409267	-0,19392
MLLT6	1,162450917	0,329729	0,793092491	1,537141048	0,010086	0,02351341	0,00243065	-0,24042
EBF1	1,172499296	0,047785	0,159108146	1,197752542	0,031562	0,024346352	0,02081873	-0,17335
PAQR7	1,271563973	0,078017	0,250720547	1,423414357	0,013182	0,025563879	0,02370218	-0,19128
ORAI3	1,396658362	0,018871	0,00153112	1,30973614	0,066351	0,031670387	0,03121917	-0,23416
PERP	0,93116616	0,583781	0,990144411	1,382476753	0,016868	0,03343778	0,00171836	-0,27336
ARHGAP1**	1,53172734	0,042	0,014788729	1,934476577	0,002439	0,034704543	0,00258733	-0,20456
ANXA9	1,065034844	0,433775	0,163817364	1,225923885	0,025828	0,038007212	0,0008204	-0,38624
RBM26	0,951174994	0,729082	0,963217018	1,451792099	0,021897	0,039995677	0,04211576	-0,28763
ATP6V1B1**	1,272012881	4,79E-06	0,00025335	1,161398525	0,011514	0,041927803	0,02376163	-0,25146
GPX8	1,130755392	0,116592	0,40719758	1,174105198	0,049533	0,045452506	0,02589602	-0,55645
HIST4H4	1,142076123	0,146282	0,227227589	1,304481289	0,007627	0,047737512	0,02353604	-0,30325
MTIF3	0,99769224	0,985546	0,963994487	1,33420682	0,034782	0,050501158	0,03419489	-0,36718
UBTF	1,168210398	0,540831	0,567164588	1,881540446	0,018025	0,050675222	0,01467615	-0,22178
AGRN	1,409250283	0,002384	0,049602943	1,507315498	0,000554	0,06023899	0,01477528	-0,22069
OSBPL3	1,36507596	0,019864	0,026384075	1,224626845	0,159943	0,066231515	0,04521738	-0,31273
C2CD2L	1,601496678	0,013879	0,041455708	1,601473161	0,019109	0,078344555	0,0200425	-0,21981
MARVELD1	1,418864885	0,006421	0,034675802	1,376818961	0,017173	0,091488135	0,02004398	-0,28208
CNP	1,648679929	0,024382	0,041364361	1,766580912	0,012931	0,100581187	0,01253684	-0,23413
MAP2K3	1,32809589	0,042245	0,024660014	1,093360411	0,531619	0,141310969	1,532E-05	-1,84694
HIST1H2AC	1,37871549	0,000584	0,003485404	1,23077451	0,035399	0,179055949	0,00483502	-0,47969
PRKACA	1,644529646	0,039933	0,028772505	1,36859023	0,199032	0,198714997	0,03617588	-0,23991
RAB17	1,416375998	0,012536	0,028493693	1,248642508	0,122172	0,231508283	0,01185752	-0,25402
HIST1H2BD	1,439031097	7,86E-05	0,01149735	1,266580672	0,013767	0,33405993	0,03023882	-0,41073
MAN2C1	1,627476403	0,000801	0,004257794	1,331340329	0,06293	0,343069016	0,02812641	-0,4211
LRRC61	1,346604294	0,013875	0,028212069	1,086147576	0,501982	0,367541337	0,00288737	-0,33535
HIST1H4H	1,286768229	0,000482	0,032068948	1,186632063	0,027314	0,442201124	0,00595406	-0,95329
HIST2H2BF	1,268587177	0,000583	0,010004542	1,118124784	0,122847	0,505829881	0,00809903	-0,34071
ULK3	1,671161681	0,00446	0,035865966	0,903737382	0,570436	0,612375224	0,00421396	-0,21357
PYGO2	1,745206162	0,038051	0,014695678	1,656722486	0,067181	0,695046013	0,01378117	-0,20279
HIST1H3D	1,247567512	0,002617	0,035482873	1,155097918	0,058925	0,901301812	0,00055397	-0,54283
UPK3B	1,163807383	0,001909	0,048991913	1,050290757	0,3448	0,974371972	0,04895307	-0,25918

** Genes selected for real time PCR validation

Suppl. Table 2. **Upregulated Gene Signature upon MKK3 depletion.**

gene	OS			DFS				
	**HR cox univ**	***p***	**logrank p(zscore)**	**HR cox univ**	***p***	**logrank p(zscore)**	**permut p**	**log2FC**
**ALG2**	0,981052973	0,931813849	0,998555316	0,603079035	0,023404	0,025984432	0,00621	0,156515
**BCAS4**	0,956910448	0,56823963	0,459242839	0,776136176	0,002295	0,008531268	0,02597	0,352239
**ENOPH1**	0,681360941	0,061285416	0,206096528	0,461072048	0,000488	0,00343598	0,046217	0,20532
**F2RL1****	0,699020324	0,009136868	0,038154922	0,639080156	0,001313	0,00654051	0,031655	0,195627
**GLO1**	0,802229669	0,093030891	0,217921718	0,676796673	0,002244	0,000281403	0,044997	0,192403
**GSPT1****	0,522394294	0,00013395	0,000854321	0,697925698	0,068847	0,003179645	0,018949	0,262678
**IFNGR1**	0,796493545	0,106869111	0,345388249	0,605315707	0,000571	0,001085918	0,03368	0,292655
**LRRC59**	0,526768965	0,001127229	0,003025729	0,646005871	0,039736	0,081452931	0,001791	0,293636
**MAP7**	0,695419256	0,016559084	0,033261535	0,926554368	0,653371	0,941779986	0,000175	0,213039
**NAT1****	0,747279124	0,001991502	0,003289871	0,730274147	0,001227	0,000381098	0,021976	0,360377
**RIMKLA**	0,900744138	0,198636185	0,05740211	0,84845389	0,041798	0,046022325	0,009465	0,268305
**SH3D19**	0,785589652	0,041697816	0,031390296	0,865566519	0,267099	0,093685902	0,014528	0,170158
**UBE2D2**	0,542257864	0,003178051	0,03737895	0,573975631	0,009393	0,002444204	0,006251	0,212986
**UBE2K**	0,620082353	0,023969414	0,014835704	0,553984465	0,007971	0,00390235	0,009998	0,172009
**UBTD2**	0,717211049	0,027059143	0,004072699	0,867490873	0,397339	0,062866839	0,046862	0,201948
**USP7**	0,63380862	0,014293338	0,002204224	1,151606082	0,516639	0,257970854	0,016191	0,339324

**** Genes selected for real time PCR validation**

Suppl. Table 3. AT9283 NIH-LINCS scores in up- and down-regulated gene signatures.

Rank	Score	Type	ID	Name	Description
7832	-99.78	cp	BRD-K24576554	AT-9283	JAK inhibitor
97	99.88	cp	BRD-K24576554	AT-9283	JAK inhibitor
